# Effects of rainfall patterns in dry and rainy seasons on the biomass, ecostoichiometric characteristics, and NSC content of *Fraxinus malacophylla* seedlings

**DOI:** 10.3389/fpls.2024.1344717

**Published:** 2024-03-12

**Authors:** Shaojie Zheng, Xiaofei Cha, Qiong Dong, Huanxian Guo, Lijuan Sun, Qize Zhao, Yunqi Gong

**Affiliations:** ^1^ College of Forestry, Southwest Forestry University, Kunming, Yunnan, China; ^2^ Southwest Mountain Forest Resources Conservation and Utilization of the Ministry of Education, Kunming, China; ^3^ Nujiang Prefecture Forestry and Grassland Bureau, Nujiang Yunnan, China

**Keywords:** rainfall pattern, dry and rainy season, biomass, eco-stoichiometry, NSC, Fraxinus malacophylla

## Abstract

With global climate change and rising temperatures, rainfall will change. The impact of global rainfall changes on ecosystems has prompted people to delve deeper into how changes in rainfall affect plant growth; Plant biomass, nutrient element content, and non-structural carbohydrate content are very sensitive to changes in precipitation. Therefore, understanding the impact of rainfall changes on seedlings is crucial. However, it is currently unclear how the seedlings of *Fraxinus malacophylla* Hemsl in rocky desertification areas respond to changes in rainfall. In this study, the response of biomass, nutrient accumulation, and NSC content of *Fraxinus malacophylla* Hemsl seedlings to different rainfall intervals and rainfall during the dry and rainy seasons was studied. Use natural rainfall duration of 5 days (T) and extended rainfall duration of 10 days(T_+_) as rainfall intervals; average monthly rainfall was used as the control (W), with a corresponding 40% increase in rainfall (W_+_) and a 40% decrease in rainfall (W_-_) as rainfall treatments. The research results indicate that the biomass of roots, stems, and leaves, as well as the accumulation of C, N, and P in *Fraxinus malacophylla* Hemsl seedlings increase with the increase of rainfall, while the soluble sugar and starch content show a pattern of first increasing and then decreasing. The biomass and nutrient accumulation of each organ showed root>leaf>stem. Except for the beginning of the dry season, prolonging the duration of rainfall in other periods inhibits the biomass accumulation of *Fraxinus malacophylla* Hemsl seedlings, and promotes the accumulation of C, N, and P nutrients and an increase in soluble sugar and starch content. There was a significant positive correlation (*P*<0.05) between the nutrient contents of C, N, and P in various organs, as well as between soluble sugar and starch content; And N: P>16, plant growth is limited by P element. These results indicate that changes in rainfall can affect the growth and development of *Fraxinus malacophylla* Hemsl seedlings, increasing rainfall can promote biomass and nutrient accumulation of *Fraxinus malacophylla* Hemsl seedlings, and prolonging rainfall intervals and reducing rainfall have inhibitory effects on them. The exploration of the adaptation of *Fraxinus malacophylla* Hemsl seedlings to rainfall patterns has promoted a basic understanding of the impact of rainfall changes on the growth of *Fraxinus malacophylla* Hemsl. This provides a theoretical basis for understanding how *Fraxinus malacophylla* Hemsl can grow better under rainfall changes and for future management of *Fraxinus malacophylla* Hemsl artificial forests in rocky desertification areas.

## Introduction

Global ecosystems are experiencing the pressure brought about by anthropogenic climate change. In the past few decades, changes in the carbon cycle and the intensification of greenhouse gas emissions have significantly influenced global climate change by human activities (Hoegh-Guldberg et al., 2019). Climate change will affect the distribution of annual and total rainfall (Goldstein and Suding, 2014). According to reports, these changes have caused the current concentration of carbon dioxide in the atmosphere to be higher than 800000 years ago (Lüthi et al., 2008). The increase in the average temperature of the world by 0.85°C compared to the pre-industrial period (Hoegh-Guldberg et al., 2019) is a cause for concern for mankind. The continuous rise in temperature will alter the intensity and process of water cycling in terrestrial ecosystems, leading to changes in global rainfall patterns (Masson-delmotte et al., 2021). Changes in rainfall can affect the effectiveness of soil moisture and nutrients, leading to changes in plant nutrient utilization and allocation strategies. This is particularly noticeable in semi-arid and arid regions where water resources are severely scarce. Extreme rainfall events (Jentsch et al., 2007; Smith, 2011a, b) or severe droughts (He et al., 2017) may lead to suppressed plant growth and rapid plant degradation.

An important manifestation of global rainfall pattern changes is the significant changes in the characteristics of rainfall events (e.g. rainfall interval, intensity, and size). Research has found that the effective utilization of plant water by rainfall is closely related to factors such as rainfall and rainfall interval (Alan et al., 2008). The impact of rainfall events on plants depends on both rainfall and rainfall intervals. The impact of high-frequency minor rainfall events and low-frequency massive rainfall events on plants has become a current research hotspot. Studies have shown that the impact of rainfall on plants depends on rainfall time intervals, and the extension of rainfall time intervals significantly promotes the accumulation of biomass due to increased rainfall (Walck et al., 2011; Vile et al., 2012). Fay (2000) believes that changes in rainfall intervals may be the main factor affecting aboveground net primary productivity; When the total precipitation remains unchanged, changing the frequency and intensity of rainfall may be an important factor in regulating plant productivity (Post and Knapp, 2020). In ecosystems, the distribution of rainfall during the growing season (including the size and frequency of rainfall events and the length of drought intervals) has a more significant impact on plant biomass, coverage, and species diversity than the total precipitation; This is because the distribution of rainfall directly affects the absorption of soil moisture by plants (Loik et al., 2004). Yue et al. (2016) found that low-frequency massive rainfall events were more effective in increasing the biomass of annual plants than high-frequency minor rainfall events while maintaining the total precipitation unchanged. In ecosystems, the main source of water for plants is natural rainfall (Sponseller, 2007); The root system is the organ that first perceives changes in environmental moisture, then transmits chemical signals upwards to regulate stomatal behavior (Jia and Zhang, 2008), and adapt to different water environments by adjusting its own morphology and biomass. At present, a large number of studies on the impacts of changing rainfall patterns on terrestrial ecosystems have been carried out by ecologists around the globe, covering a wide range of aspects such as plant growth, species composition, biodiversity, community structure, and characteristics (Knapp et al., 2002; Jia and Zhang, 2008; Kimball et al., 2010; Worm and Lotze, 2021). In terms of plant growth, the impact of changes in rainfall patterns on the allocation of seedling biomass has received widespread attention; Previous studies have shown that changes in rainfall patterns can significantly alter the distribution of root biomass in plants; Increasing rainfall can promote the accumulation of plant biomass (Fay et al., 2003), while extending rainfall intervals can inhibit the accumulation of plant root biomass (Harper et al., 2005). Therefore, strengthening the research on the impact of rainfall pattern changes on plant growth and biomass is of great significance for understanding the impact of climate change on plant growth.

Ecological stoichiometry refers to the balance of various elements in an ecosystem, especially the stable state between carbon (C), nitrogen (N), and phosphorus (P), which is crucial. Since the introduction of ecological stoichiometry, it has been used for research on nutrient dynamics (Zechmeister et al., 2015), biogeochemical cycles (Wurzburger and Wright, 2015), and climate change response mechanisms (Sun et al., 2021). Carbon, nitrogen, and phosphorus are essential nutrients for plant growth, and the measurement values of C, N, and P are important indicators determining plant nutrient limitations (Wang et al., 2013; Mao et al., 2016). The ratio of C: N and C: P can reflect the growth rate and nutrient utilization rate of plants; Meanwhile, the N: P value of plant leaves can be used as a threshold for plant nutrient limitation (Yuan et al., 2011; Yang et al., 2021). The interaction between carbon, nitrogen, and phosphorus occurs in terrestrial ecosystems. Plant growth is essentially a process of accumulating elements (mainly C, N, and P) and adjusting their relative proportions to maintain their own stability (Dai et al., 2022). The ecological stoichiometry theory suggests that organisms have the ability to maintain a relatively stable composition of chemical elements in their bodies during the process of adapting to environmental changes, and the element content or ratio remains relatively stable within a certain range with environmental changes (Elser et al., 2010). However, the contents and proportions of different elements vary among organs (Ma et al., 2017) and respond accordingly to changes in the external environment (Li et al., 2017). It has been found that C provides a certain material basis for plants (Martin et al., 2018), while N is the main component of proteins and plays a crucial role in plant photosynthesis, growth, and decomposition of dead branches and leaves (Klausmeier et al., 2004; Yang et al., 2018); P is a component of DNA and RNA, primarily responsible for the structure of plant cells, and enhances the assimilation of C or N (Elser et al., 2000; Sardans et al., 2012). The content of these elements and their ecological stoichiometric characteristics can reflect the internal stability of plant organs, as well as the distribution ratios and interrelationships of various elements in different organs (Liu et al., 2019; Luo et al., 2021). By studying the ecological stoichiometric characteristics of plant organs, it is possible to understand nutrient cycling and utilization during plant growth, breeding, regeneration, and restoration processes. Research has shown that there is a certain correlation between the ecological stoichiometric characteristics of plant roots, stems, and leaves at the individual level (Zhang et al., 2018). Whereas, an increase or decrease in rainfall affects soil pH and cation exchange capacity, which in turn affects plant nutrient accumulation and stoichiometry (Sardans et al., 2012; Thakur et al., 2015; Sierra et al., 2017; Prommer et al., 2019). In addition, the specific effects of rainfall changes and rainfall intervals on the stoichiometric ratios of various organs of *Fraxinus malacophylla* Hemsl seedlings have not been clarified. Therefore, the study of the ecological stoichiometric characteristics of carbon, nitrogen, and phosphorus in *Fraxinus malacophylla* Hemsl seedlings will help to understand the growth regulation mechanisms and survival strategies of *Fraxinus malacophylla* Hemsl seedlings.

The main components of non-structural carbohydrates (NSCs) are soluble sugars and starch. It is one of the main components of soil active organic matter (Wu et al., 2019; Xie et al., 2022) and is also a product of photosynthesis and respiration in plants. Its content can determine the carbon supply and demand status in plants (Wang et al., 2021). Soluble sugars are the main form of carbohydrate transport and utilization in plants, playing an important role in maintaining cell osmolality pressure and resisting stress; Starch is the main storage substance in plants, and the two can be converted into each other under certain conditions (Latt et al., 2001). The content and distribution of NSC affect plant growth and adaptation strategies to the environment (Loewe et al., 2000). Hence, the study of plant NSC characteristics has become a hot topic reflecting the adaptability to environmental changes (Myers and Kitajima, 2007). Research on plant NSC mainly focuses on the allocation patterns of NSC (Yin et al., 2009; Yu et al., 2011), spatial-temporal distribution characteristics (Zhang et al., 2015), and response to environmental changes, such as nitrogen and phosphorus addition (Wang et al., 2014), drought stress (Du et al., 2014), CO_2_ doubling (Dong et al., 2015), and high-temperature stress (Li N. et al., 2014); However, there is limited exploration of rainfall changes (Zheng et al., 2014). Studies have shown that water changes have a significant impact on the content and composition of NSC in plants (Yang et al., 2019); Goodsman et al. (2010) discovered that nitrogen addition led to a decrease in starch content in the roots of Pinus tabulaeformis. McDowell (2011) found that when water is scarce, plant photosynthesis is lower than respiration, and NSC content decreases. Changes in precipitation and precipitation regions, and frequent occurrence of extreme climate events, will further affect the global environment and ecosystem functions (Wang et al., 2005). Therefore, studying the changes in plant NSC content and its distribution in different organs in the context of global climate change helps to understand the impact of climate change on plant growth and the response and adaptation mechanisms of physiological ecological processes. It is of great significance to conduct research on the impact of rainfall changes on the storage, distribution, and transformation of plant NSC.

*Fraxinus malacophylla* Hemsl is a semi-deciduous tree belonging to the genus *Fraxinus* of Oleaceae, which is mainly distributed in Alpine mixed forests in karst areas of Guangxi and Yunnan. *Fraxinus malacophylla* Hemsl has good adaptability to rocky desertification areas, a high survival rate of afforestation, and is an excellent broad-leaved tree companion species in rocky desertification management with high medicinal and ecological value (Li, 2005). Mainly used for treating constipation, epilepsy, malaria, and manufacturing furniture (Tan et al., 2013; Huang and Chen, 2014). As one of the main native species, *Fraxinus malacophylla* Hemsl is also widely used in the ecological restoration of rocky desertification areas. At present, numerous scholars have done systematic studies on the germination characteristics (He et al., 2012), fertilization management (Xia et al., 2016), and drought resistance (Gao et al., 2015) of *Fraxinus malacophylla* Hemsl seeds. However, in rock-deserted areas, rainfall has obvious seasonal variations, e.g. Yunnan generally shows a pattern of low precipitation in spring and winter and high precipitation in summer and autumn, and during ecological restoration, *Fraxinus malacophylla* Hemsl will face drought habitats (Tan et al., 2011). Nevertheless, the effects of increased or decreased rainfall and the duration of rainfall intervals on the biomass, ecological stoichiometric characteristics, and NSC content of various organs of *Fraxinus malacophylla* Hemsl seedlings are not yet clear, and the relationship between different nutrient elements in the same organ is not yet clear. Therefore, this study aims to monitor the dynamic changes in biomass, ecological stoichiometric characteristics, and NSC content of various organs of the *Fraxinus malacophylla* Hemsl seedlings by simulating the impact of rainfall patterns during the dry and rainy seasons on their growth; To provide a theoretical basis for plants to adapt to global climate change and make certain growth regulation mechanisms and survival strategies in specific regions, as well as to understand how *Fraxinus malacophylla* Hemsl grow in climate change. For this reason, we assume that: (1) increasing rainfall increases the biomass, and nutrient accumulation, and NSC content of the *Fraxinus malacophylla* Hemsl seedlings. (2) Extending the interval between rainfall inhibited the growth of *Fraxinus malacophylla* Hemsl seedlings. (3) The biomass, nutrient accumulation, and NSC content of various organs in the *Fraxinus malacophylla* Hemsl seedling showed root>leaf>stem. (4) Under the rainfall pattern, there is a significant positive correlation between C and N, C and P, and N and P. This study aims to answer the following questions: (1) How does increasing or decreasing rainfall affect the biomass, ecological stoichiometry, and changes in NSC content of *Fraxinus malacophylla* Hemsl seedlings; Is there a phenomenon of promoting rainfall and suppressing rainfall reduction? (2) How do changes in biomass, ecological stoichiometry, and NSC content of *Fraxinus malacophylla* Hemsl seedlings adapt to rainfall duration, and whether prolonging rainfall duration promotes or suppresses it? (3) What is the relationship between C, N, and P in the same organ of *Fraxinus malacophylla* Hemsl seedlings under rainfall patterns, and is it a significant positive correlation or a negative correlation?

## Materials and methods

### Plant material and treatment

The research was conducted in a greenhouse at Southwest Forestry University of China (Kunming, Yunnan, 25° 03 ′ N, 102° 46 ′ E). The research area is located in the subtropical plateau monsoon climate zone, with an average elevation of 1954 meters, few frost periods, and a warm climate; The annual average temperature is 16.5 °C, the annual precipitation is 1035mm, the relative humidity of the air is 23%-67%, the atmospheric CO_2_ concentration is 400-412 ppm, and there is sufficient light.

The plant material used was 2-year-old seedlings of *Fraxinus malacophylla* Hemsl supplied by Yunnan Jianshui Chengfa Greening Co., Ltd. On June 8, 2022, the seedlings were taken from Southwest Forestry University. After 90 days of refining, the seedlings were transplanted into containers with an upper diameter of 20cm, a lower diameter of 14cm, and a height of 18cm. They were adapted for growth in the greenhouse. Select one well-growing seedling from each pot for planting, and use a mixture of red soil, humus soil, and perlite in a ratio of 5:3:2 as the test soil for potted soil; Its field capacity is 26.43%, pH value is 5.52, bulk density is 1.31g/cm^3^, soil organic matter is 3.23g/kg, organic carbon is 32.43g/kg, total nitrogen is 0.86g/kg, total phosphorus is 0.41g/kg, hydrolyzed nitrogen is 45.62mg/kg, and available phosphorus is 11.78mg/kg.

### Experimental design

A two-factor randomized block trial was used in this study. Factor 1 is the rainfall interval, according to Yunnan Province in southwestern China, the annual average number of consecutive days without effective precipitation is about 5 days (Liu et al., 2020). Therefore, taking the natural rainfall duration of 5 days and the extended rainfall duration of 10 days as rainfall intervals, they are respectively denoted as T and T_+_. Factor 2 is rainfall. According to Duan et al. (2019) on meteorological data and Li (2016) On the spatiotemporal distribution of rainfall, the average annual rainfall in Kunming, Yunnan from 1988 to 2017 was 975.5mm, with the highest rainfall year being 1449.9mm in 1999, which is 45% higher than in previous years; The minimum number of children was 565.8 mm in 2009, which is 42% less than previous years; The maximum annual precipitation is 1.30-1.60 times the average annual precipitation, and the minimum annual precipitation is 0.47-0.71 times the average annual precipitation. Therefore, with the monthly average rainfall as the control (W), corresponding rainfall increases of 40% (W_+_) and decreases of 40% (W_-_) are used as rainfall treatments. The specific rainfall is shown in **Table 1**. The experiment consists of 6 treatments, each with 3 zones, with 16 seedlings in each zone, totaling 288 plants. Water control research was conducted from October 28, 2022, to September 28, 2023, after adapting to growth for 50 days in the Greenhouse.

**Table 1:** Rainfall design.

Month	Monthly average rainfall/mm	Precipitation interval/d	Monthly watering frequency	Single watering amount/ml		
				W_-_	W	W_+_
November to January (At the beginning of the dry season)	25.41	T T_+_	6 3	80 160	133 266	186 372
February to April (At the end of the dry season)	20.34	T T_+_	6 3	63 127	106 212	148 297
May to July (At the beginning of the rainy season)	146.76	T T_+_	6 3	460 921	768 1536	1075 2150
August to October (At the end of the rainy season)	150.06	T T_+_	6 3	471 942	785 1570	1099 2199

### Sample determination and method

In December 2022 March, June, and September 2023, 3 seedlings were randomly selected from each treatment, totaling 12 seedlings. Wash with clean water and filter paper to absorb moisture. Place each organ in an envelope and mark it before taking it back to the laboratory to obtain fresh weight. Put it in an oven with a temperature of 105 °C and sterilize it for 30 minutes, then adjust it to 80 °C and dry it to constant weight. Weigh each organ to get the biomass of roots, stems, and leaves and the biomass of a single plant (g). We also calculated leaf moisture content = (leaf fresh weight-dry weight)/dry weight, degree of fleshing = leaf fresh weight/dry weight, and biomass allocation of roots, stems, and leaves. Grind the sample into 0.15mm powder using a grinder to determine the nutrient content of C, N, and P in each organ. The biomass expressed in the text is the dry weight of each organ of the *Fraxinus malacophylla* Hemsl.

Organic carbon was determined using the potassium dichromate sulfuric acid oxidation external heating method, total nitrogen was determined using the Kjeldahl digestion method, and total phosphorus was determined using the NaOH alkali solubilization molybdenum antimony resistance colorimetric method; The specific measurement method is described in detail in (Qu et al., 2014). The product of nutrient content and corresponding biomass is the accumulation of C, N, and P (mg), and the sum of the accumulation of C, N, and P organs is the accumulation of C, N, and P per plant. The nutrient allocation ratio is calculated based on nutrient accumulation and individual plant accumulation (Gao et al., 2021; Rastetter et al., 2022). Measure the soluble sugar and starch content (mg/g) of each organ by the phenol sulfuric method (Blumstein and Hopkins, 2021). The sum of soluble sugar and starch is the NSC content.

### Statistical analysis

Perform principal components analysis on the biomass, C, N, P, soluble sugar, and starch content of each organ. Use the power function y=ax^b^ to perform non-linear curve-fitting on the accumulation of C, N, and P in the same organ (Witek-Krowiak et al., 2011), thereby indicating the correlation between nutrient elements. When analyzing the relationship between allometric growth, convert the power function to log y=log β+α log x, where α is the allotrophic growth index (equation slope), β is the regression constant, and x and y are the biomass of the roots, stems, and leaves of *Fraxinus malacophylla* Hemsl seedlings; And the slope of the equation was obtained using Standardized Major Axis Estimation (SMA) (Warton and Weber, 2002), which was calculated using (S) MATR Version 2.0 (Falster et al., 2006). Comparing the slope of the equation with the theoretical value of 1.0, when estimating whether there is a significant difference between the slope SMA and the theoretical value of 1.0 if it exists (*P__1.0_* <0.05), it indicates that the allometric growth relationship between different organ biomass is allometric growth. If there is no significance (*P__1.0_* >0.05), it is isometric growth (Zhang W.P. et al., 2020).

Data collation using Excel 2010, and statistical analysis using SPSS 25.0 (SPSS Inc., Chicago, IL, USA), Origin 2021 (OriginLab Co., Northampton, MA, USA), and MATR Version 2.0.

## Results

### Characteristics of dry and fresh weight of each organ

#### Fresh weight content

As shown in **Table 2**, the fresh weight of roots stems, and leaves under the rainfall pattern (rainfall duration and rainfall) showed significant differences during the dry and rainy seasons (*P*<0.05). There was no significant difference (*P*>0.05) between the interaction of rainfall duration and rainfall amount for all organs at the end of the dry season (March). During the dry season period, there were no significant changes in leaf moisture content and degree of fleshing under the rainfall pattern; During the rainy season period, there was a significant difference only in the duration of rainfall (*P*<0.01). Overall, the fresh weight in the rainy season is higher than that in the dry season. The increase in rainfall promotes the accumulation of fresh weight in roots, stems, and leaves, and the duration of extended rainfall was higher than the duration of natural rainfall. The reason is that repeated rainfall can easily lead to flooding of the *Fraxinus malacophylla* Hemsl, inhibiting plant growth, and causing natural rainfall duration to be lower than the fresh weight of extended rainfall duration. At the same time, it is also clear that the seedlings of the *Fraxinus malacophylla* Hemsl can adapt to certain drought conditions.

**Table 2:** Effects of rainfall patterns on changes in fresh weight of various organs in *Fraxinus malacophylla* hemsl seedlings.

Month	Precipitation interval	Precipitation	Root	Stem	Leaf	Leaf moisture content	Degree of fleshing
December	5d	W_-_	9.54 ± 0.20c	5.10 ± 0.22c	6.51 ± 0.17c	23.33 ± 4.56a	1.23 ± 0.05ab
		W	15.09 ± 1.43b	7.11 ± 0.28b	8.87 ± 0.43b	13.96 ± 5.74b	1.14 ± 0.06b
		W_+_	22.07 ± 1.10a	11.56 ± 0.41a	12.78 ± 1.08a	24.32 ± 3.99a	1.24 ± 0.04a
	10d	W_-_	11.38 ± 0.55c	5.44 ± 0.61c	5.88 ± 0.65c	15.72 ± 4.75b	1.16 ± 0.05b
		W	18.07 ± 0.45b	10.54 ± 0.35b	7.55 ± 0.42b	23.98 ± 3.55a	1.24 ± 0.04a
		W_+_	22.81 ± 0.45a	9.37 ± 0.31a	9.86 ± 0.55a	23.67 ± 3.34a	1.24 ± 0.03a
	F value	D	23.111^**^	8.512^*^	30.97^**^	0.080^ns^	0.080^ns^
		R	322.842^**^	288.074^**^	104.574^**^	2.356^ns^	2.356^ns^
		DR	2.838^ns^	81.042^**^	5.394^*^	6.114^*^	6.114^*^
March	5d	W_-_	9.54 ± 0.20c	5.10 ± 0.22c	6.51 ± 0.17c	23.33 ± 4.56ab	1.23 ± 0.05ab
		W	15.09 ± 1.43b	7.11 ± 0.28b	8.87 ± 0.43b	13.96 ± 5.74b	1.14 ± 0.06b
		W_+_	22.07 ± 1.10a	11.56 ± 0.41a	12.78 ± 1.08a	24.32 ± 3.99a	1.24 ± 0.04a
	10d	W_-_	11.38 ± 0.55c	5.44 ± 0.61c	5.88 ± 0.65c	15.72 ± 4.75b	1.16 ± 0.05b
		W	18.07 ± 0.45b	10.54 ± 0.35a	7.55 ± 0.42b	23.98 ± 3.55a	1.24 ± 0.04a
		W_+_	22.81 ± 0.45a	9.37 ± 0.31b	9.86 ± 0.55a	23.67 ± 3.34a	1.24 ± 0.03a
	F value	D	23.111**	8.512*	30.970**	0.080ns	0.080ns
		R	322.842**	288.074**	104.574**	2.356ns	2.356ns
		DR	2.838ns	81.042ns	5.394ns	6.114*	6.114*
June	5d	W_-_	14.58 ± 1.00c	13.88 ± 0.21c	11.27 ± 0.38c	37.19 ± 2.82ab	1.37 ± 0.03ab
		W	28.62 ± 1.27b	17.08 ± 0.86b	13.9 ± 0.22b	30.21 ± 4.62b	1.30 ± 0.05b
		W_+_	38.63 ± 1.18a	22.07 ± 0.92a	17.06 ± 0.07a	40.01 ± 3.48a	1.40 ± 0.03a
	10d	W_-_	16.10 ± 0.31c	11.13 ± 0.01c	10.48 ± 0.92b	44.22 ± 5.46a	1.44 ± 0.05a
		W	29.79 ± 0.52b	15.45 ± 1.24b	11.71 ± 0.52b	51.48 ± 4.52a	1.51 ± 0.05a
		W_+_	39.32 ± 0.26a	17.78 ± 0.39a	13.45 ± 0.67a	47.48 ± 6.73a	1.47 ± 0.07a
	F value	D	7.720^*^	68.187^**^	73.527^**^	28.003^**^	28.003^**^
		R	1146.842^**^	149.746^**^	98.276^**^	0.774^ns^	0.774^ns^
		DR	0.352^ns^	4.881^*^	10.082^**^	4.309^*^	4.309^*^
September	5d	W_-_	26.66 ± 2.00c	13.88 ± 0.21c	13.84 ± 0.55c	41.88 ± 1.94a	1.42 ± 0.02a
		W	37.62 ± 2.14b	17.08 ± 0.86b	16.89 ± 0.50b	44.29 ± 11.75a	1.44 ± 0.12a
		W_+_	42.15 ± 0.58a	22.07 ± 0.92a	19.86 ± 0.77a	35.47 ± 3.18a	1.35 ± 0.03a
	10d	W_-_	33.47 ± 1.29c	11.13 ± 0.01c	13.04 ± 0.12c	51.66 ± 7.59a	1.52 ± 0.08a
		W	35.57 ± 0.54b	15.45 ± 1.24b	15.61 ± 0.42b	56.67 ± 5.18a	1.57 ± 0.05a
		W_+_	43.85 ± 0.56a	17.78 ± 0.39a	19.39 ± 0.89a	59.49 ± 2.94a	1.59 ± 0.03a
	F value	D	11.154^**^	68.187^**^	9.905^*^	26.104^**^	26.104^**^
		R	134.425^**^	149.746^**^	161.723^**^	0.570^ns^	0.570^ns^
		DR	15.902^**^	4.881^*^	0.710^ns^	2.112^ns^	2.112^ns^

The data in the table is the mean ± standard deviation. Different lowercase letters in the same column indicate significant differences in rainfall duration among different rainfall levels (P<0.05). D represents the precipitation interval, R represents rainfall, and D × R is the interaction between rainfall interval and rainfall. Ns: P>0.05, *P<0.05, **P<0.01. The same as below.

#### Change in dry weight

**Figure 1:**
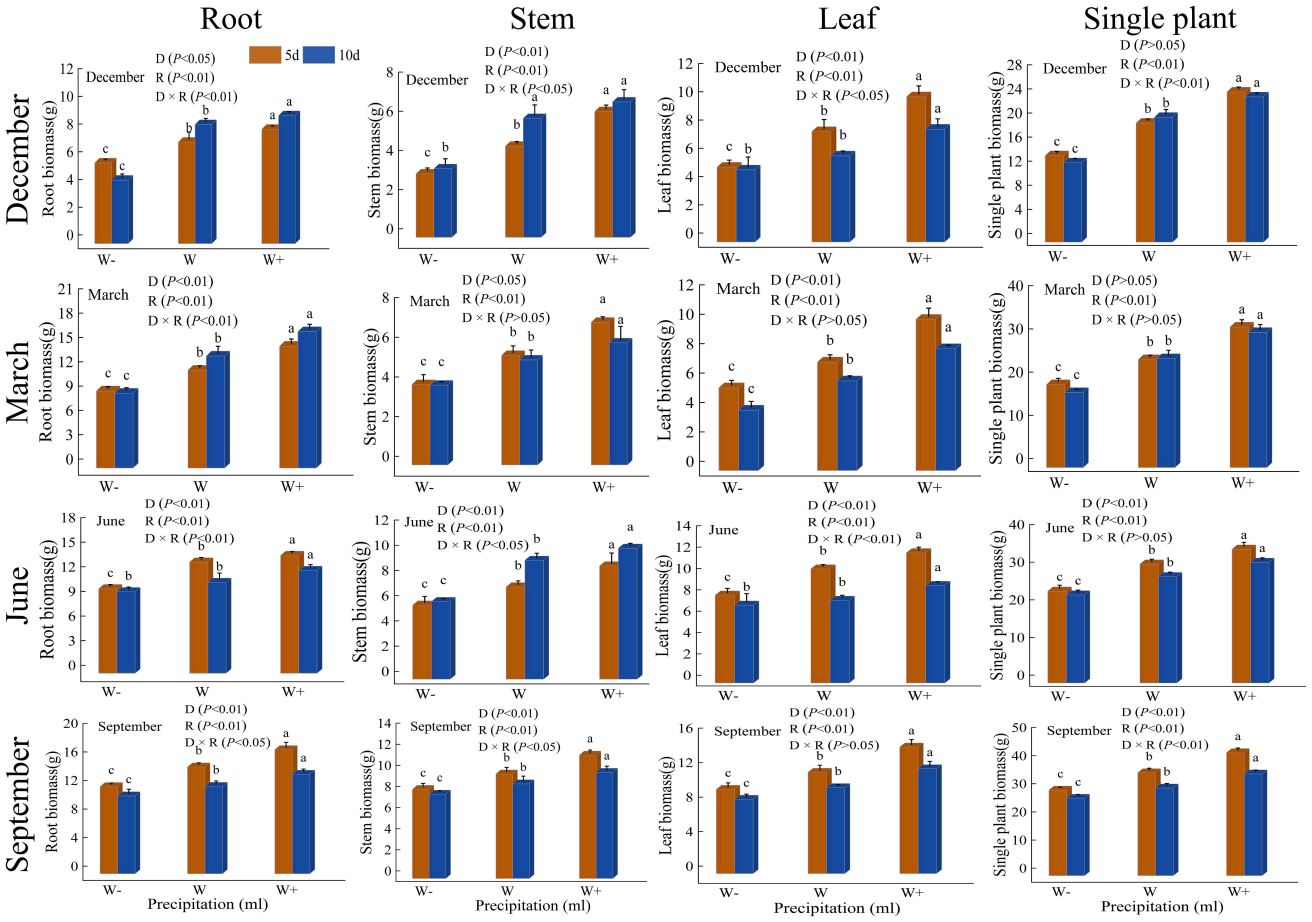
The impact of rainfall patterns on the biomass changes of various organs in the *Fraxinus malacophylla* Hemsl. Different lowercase letters indicate significant differences among different rainfall levels under the same rainfall duration, *P*<0.05. The same as below.

From **Figure 1**, it can be seen that there is a significant difference (*P*<0.05) in the rainfall pattern (rainfall duration and rainfall) on the biomass of various organs of the *Fraxinus malacophylla* Hemsl, and there is both significant and no significant difference under the interaction between the two. At the beginning of the dry season (December), except for leaf biomass, the root and stem biomass showed higher levels of 10-day rainfall treatment than 5-day rainfall. At the end of the dry season, this was only the case for root biomass. During the rainy season, the biomass of each organ showed a higher 5-day rainfall than a 10-day rainfall (except for June leaves). From the perspective of individual plant biomass, at the beginning of the dry season, the 5-day increase in rainfall was significantly higher than normal rainfall by 26.15%, the reduction in rainfall treatment was significantly lower than 37.77%, and the 10-day increase in rainfall increased by 16.30%. At the end of the dry season, the 5-day increase in rainfall is 1.30 times that of natural rainfall, while the 10-day increase in rainfall is 1.24 times. At the beginning of the rainy season, the 5-day increase in rainfall was 3.98g higher than the natural rainfall, and the reduction in rainfall treatment was 7.16g. The 10-day increase in rainfall was 3.80g higher, and the reduction in rainfall treatment was 4.86g. At the end of the rainy season, the biomass of a single plant increased by 5 days of rainfall was 43.71g, and by 10 days of rainfall, it was 36.12g. The above indicates that increasing rainfall treatment significantly promotes the accumulation of biomass in *Fraxinus malacophylla* Hemsl, while reducing rainfall treatment has an inhibitory effect, and the 5-day rainfall duration is better than the 10-day rainfall duration. The overall biomass of each organ showed root>leaf>stem, which may be due to a greater emphasis on the accumulation of root biomass during plant growth to improve the ability to absorb water and nutrients, thereby increasing the chances of survival.

There are certain significant differences in the allocation of biomass in the roots, stems, and leaves of *Fraxinus malacophylla* Hemsl between the dry and rainy season rainfall patterns (**Table 3**). At the beginning of the dry season, there was no significant difference in rainfall duration only in the allocation of root biomass, while there was a significant difference in stem and leaf biomass (*P*<0.01). However, different rainfall treatments were exactly the opposite. In the interaction between the two, except for the stem, there was no significant difference, all other significant differences were observed. At the end of the dry season and the beginning of the rainy season, there is only a significant difference in the proportion of stem biomass under the influence of rainfall duration and rainfall. There was no significant difference in the root, stem, and leaf biomass percentage at the end of the rainy season under both interactions.

**Table 3:** Analysis of variance in biomass allocation of various organs of *Fraxinus malacophylla* hemsl seedlings under rainfall pattern.

Month	Handle	Root	Stem	Leaf
December	D	ns	**	**
	R	*	ns	ns
	DR	**	ns	**
March	D	**	**	**
	R	*	ns	*
	DR	ns	**	ns
June	D	ns	**	**
	R	ns	**	ns
	DR	ns	**	ns
September	D	*	*	ns
	R	ns	ns	*
	DR	ns	ns	ns

ns represents P>0.05, *Indicates P<0.05, ** indicates P<0.01.

**Figure 2:**
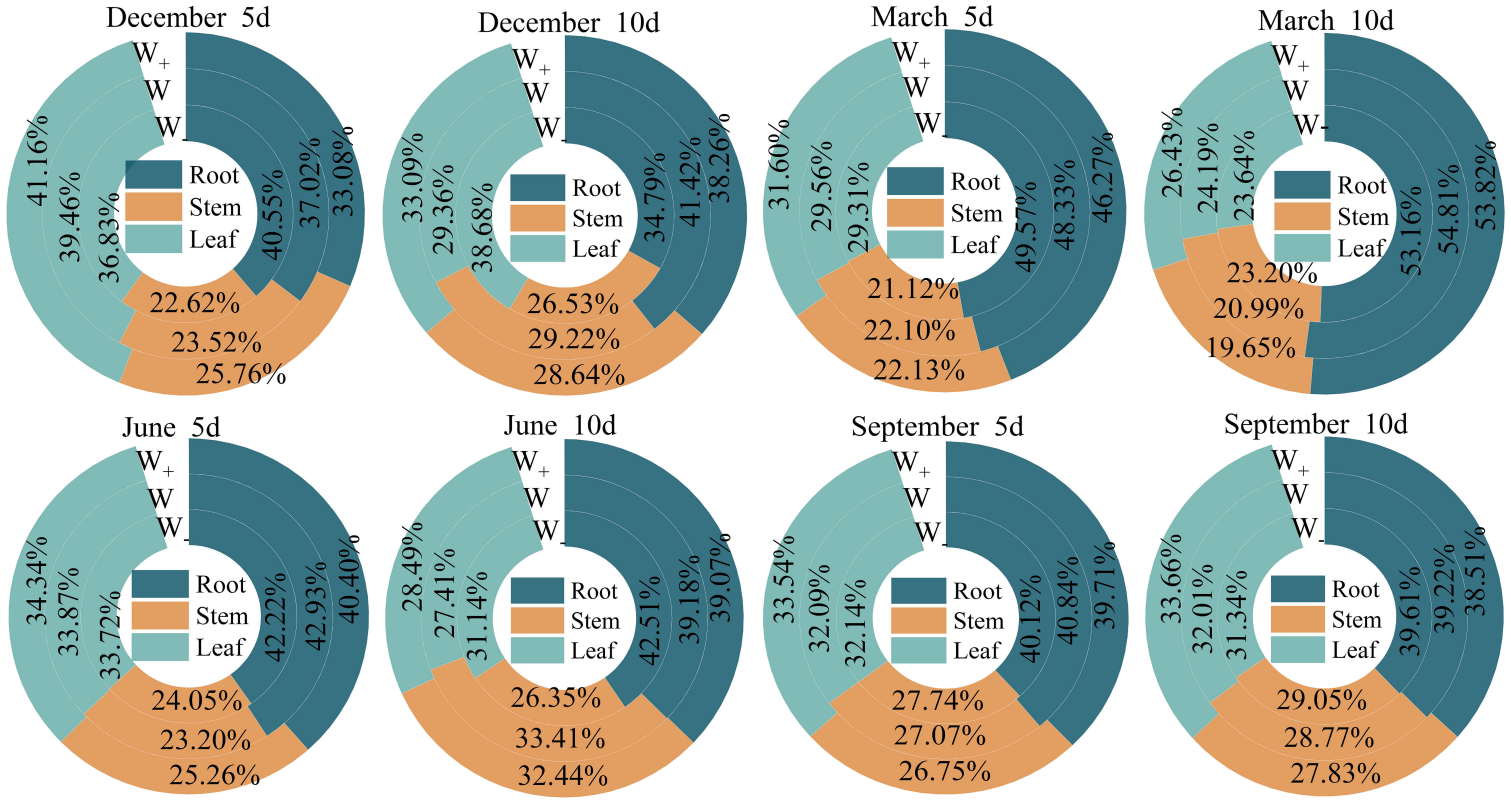
The proportion of biomass in various organs of the *Fraxinus malacophylla* Hemsl under rainfall patterns.

The impact of the proportion of biomass in each organ of the *Fraxinus malacophylla* Hemsl on the rainfall pattern is different (**Figure 2**). The allocation of root, stem, and leaf biomass in the rainy season is significantly higher than that in the dry season, and the proportion of stem and leaf biomass shows an upward trend with the increase in rainfall. Under the 10-day rainfall treatment at the end of the dry season, the root biomass accounted for the highest proportion, with a value of 53.16%; Perhaps due to drought, plants allocate more nutrients to their roots in order to withstand drought, resulting in a well-developed root system, which facilitates the extraction of water from deeper levels in the soil. In summary, there are differences in the allocation of biomass in the root, stem, and leaf of *Fraxinus malacophylla* Hemsl under different rainfall patterns, and the overall performance is as follows: root>leaf>stem, and plants are more inclined to accumulate root biomass.

### Nutrient characteristics of roots, stems, and leaves

#### Accumulation of C, N, and P

**Figure 3:**
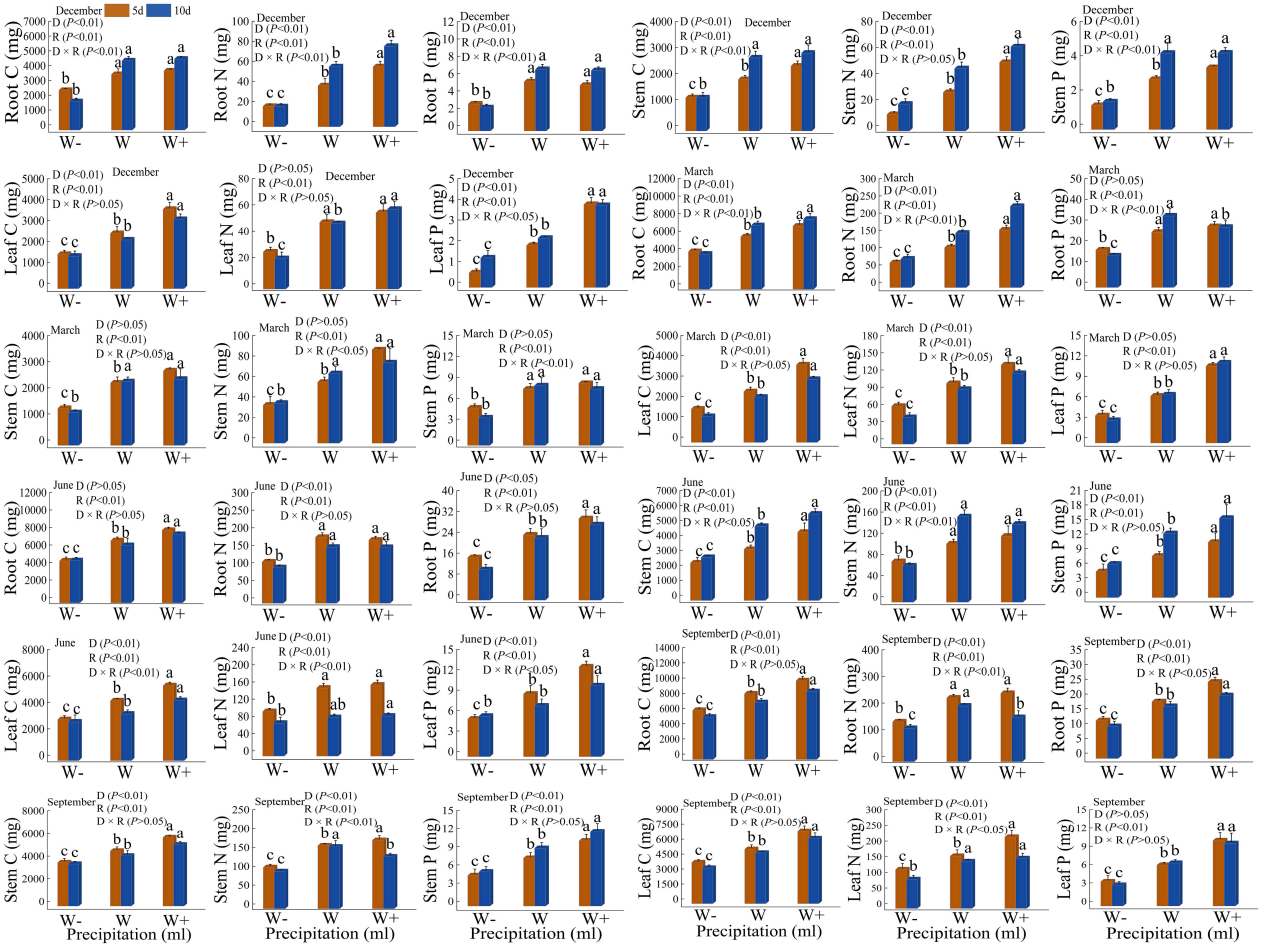
Impact of rainfall pattern on nutrient accumulation in roots, stems, and leaves of *Fraxinus malacophylla* Hemsl.

There are differences in the accumulation of carbon, nitrogen, and phosphorus in the roots, stems, and leaves of *Fraxinus malacophylla* Hemsl under different rainfall patterns (**Figure 3**). Whether in the dry or rainy season, the accumulation of carbon, nitrogen, and phosphorus in various organs significantly increased with the increase of rainfall (*P*<0.05), and increased rainfall significantly promoted the accumulation of carbon, nitrogen, and phosphorus in the *Fraxinus malacophylla* Hemsl. Under the interaction of rainfall duration and rainfall, both *P*<0.05 and *P*>0.05 existed in nutrient accumulation in each organ. At the beginning of the dry season, the accumulation of carbon, nitrogen, and phosphorus in roots, stems, and leaves under 10 days of rainfall is higher than that under 5 days of rainfall (excluding leaf carbon accumulation), indicating that under long-term drought, plants increase nutrient concentration through homeostasis mechanisms, thereby enhancing their resistance. At the end of the dry season, the accumulation of carbon, nitrogen, and phosphorus in plant stems versus carbon and nitrogen in leaves showed a higher 5 days rainfall than 10 days rainfall, while the opposite was true for carbon, nitrogen, and phosphorus of root versus phosphorus of leaf. During the rainy season, except for the carbon and nitrogen accumulation in plant stems at the beginning of the rainy season and the phosphorus accumulation in stems at the end of the rainy season, the accumulation of C, N, and P in other organs showed higher levels of short-term rainfall (5 days) than long-term rainfall (10 days); It may be that a large amount of water contains sufficient oxygen, which promotes root absorption after watering, thereby promoting plant growth. Moreover, during the rainy season, the air humidity is high and the air circulation is good, which can promote plant growth in such an environment. In conclusion, under the rainfall pattern, the nutrient accumulation in each organ shows carbon >nitrogen >phosphorus; Moreover, increasing rainfall and short-term rainfall promote the growth of *Fraxinus malacophylla* Hemsl plants, thereby increasing the accumulation of C, N, and P nutrients.

#### Metering ratio of C, N, P

**Figure 4:**
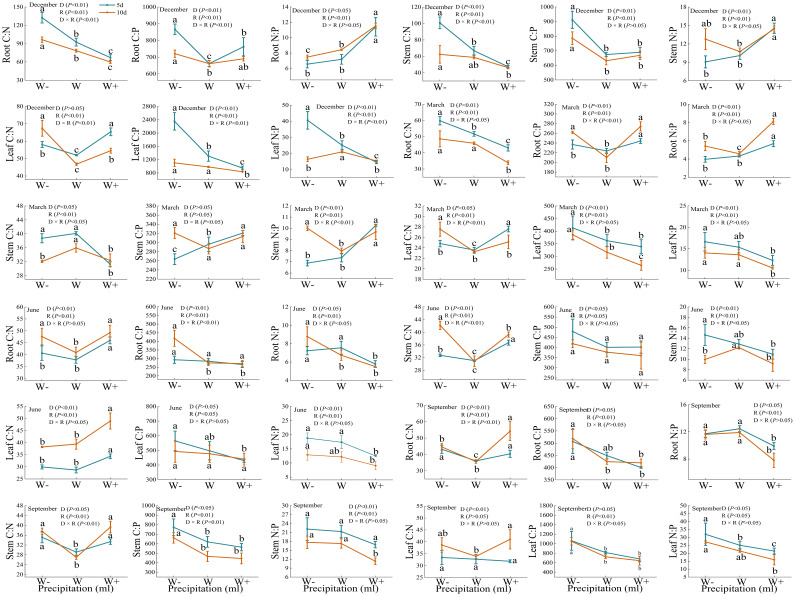
Stoichiometric ratio of roots, stems, and leaves of *Fraxinus malacophylla* Hemsl under rainfall pattern.

There are certain differences in the carbon, nitrogen, and phosphorus stoichiometry of roots, stems, and leaves under different rainfall patterns (**Figure 4**). During the dry season, except for the C: P of the stem and the C: N of the leaves, there were significant differences in the C: P, C: N, and N: P of each organ under rainfall duration and treatment (*P*<0.01). And N:P of *Fraxinus malacophylla* Hemsl leaves was greater than 16 under all rainfall treatments, indicating that the plants were limited by phosphorus in this period, and the application of phosphorus fertilizer should be emphasized when cultivating seedlings in the future. At the beginning of the rainy season (June), the C: N of the roots and stems of the *Fraxinus malacophylla* Hemsl showed a significant change pattern of first decreasing and then increasing with the increase of rainfall, while the C: P of the roots, stems and leaves showed a significant downward trend. At the end of the rainy season, except for the C: N of the root and the C: N and C: P of the stem, there was no significant difference in the stoichiometric ratios of each organ under the interaction of rainfall duration and rainfall (*P*>0.05), and the leaf N: P>16 under each treatment. In summary, the rainfall pattern during the dry and rainy seasons has a significant impact on the C, N, and P stoichiometric ratios of various organs of the *Fraxinus malacophylla* Hemsl, and the leaf N: P>16 limits plant growth due to phosphorus, which may be caused by the biological structure of the plant.

#### Nutrient allocation of roots, stems, and Leaves

From **Table 4**, it can be seen that there are certain differences in the distribution of carbon, nitrogen, and phosphorus in the roots, stems, and leaves of *Fraxinus malacophylla* Hemsl among different treatments. At the beginning of the dry season, there was no significant difference in the proportion of carbon, nitrogen, and phosphorus in plant roots versus phosphorus in plant leaves under different rainfall intervals, but there was a significant difference (*P*<0.05) under different rainfall levels. Under the interaction of the two, except for the proportion of root nitrogen, the proportion of other nutrients was significant (*P*<0.01). At the end of the dry season, there was no significant difference in the allocation of C, N, and P nutrients in various organs under the interaction between rainfall duration and rainfall (*P*>0.05), except for root carbon. At the beginning of the rainy season, there was no significant difference in the allocation of carbon and nitrogen in roots and phosphorus in leaves under different treatments, while there was a difference in carbon and nitrogen in stems and nitrogen in leaves (*P*<0.01). At the end of the rainy season, there was no significant difference in the proportion of C, N, and P allocation among various organs under the interaction of rainfall duration and rainfall (*P*>0.05).

**Table 4:** Analysis of variance for the proportion of C, N, P in various organs of *Fraxinus malacophylla* hemsl seedlings under rainfall patterns.

Month	Handle	Root			Stem			Leaf		
		C	N	P	C	N	P	C	N	P
December	D	ns	ns	ns	**	**	*	*	**	ns
	R	**	**	**	ns	*	ns	**	**	**
	DR	**	ns	**	ns	*	ns	**	ns	**
March	D	**	**	*	*	ns	ns	**	**	ns
	R	**	**	**	**	ns	ns	**	ns	**
	DR	**	ns	ns	ns	ns	ns	ns	ns	ns
June	D	ns	ns	**	**	**	**	**	**	ns
	R	ns	ns	ns	**	**	ns	ns	**	ns
	DR	ns	ns	*	**	**	ns	ns	**	ns
September	D	*	ns	**	ns	**	*	**	ns	ns
	R	ns	*	*	*	ns	ns	ns	**	**
	DR	ns	ns	ns	ns	ns	ns	ns	ns	ns

ns represents P>0.05,*Indicates P<0.05, * * indicates P<0.01.

**Figure 5:**
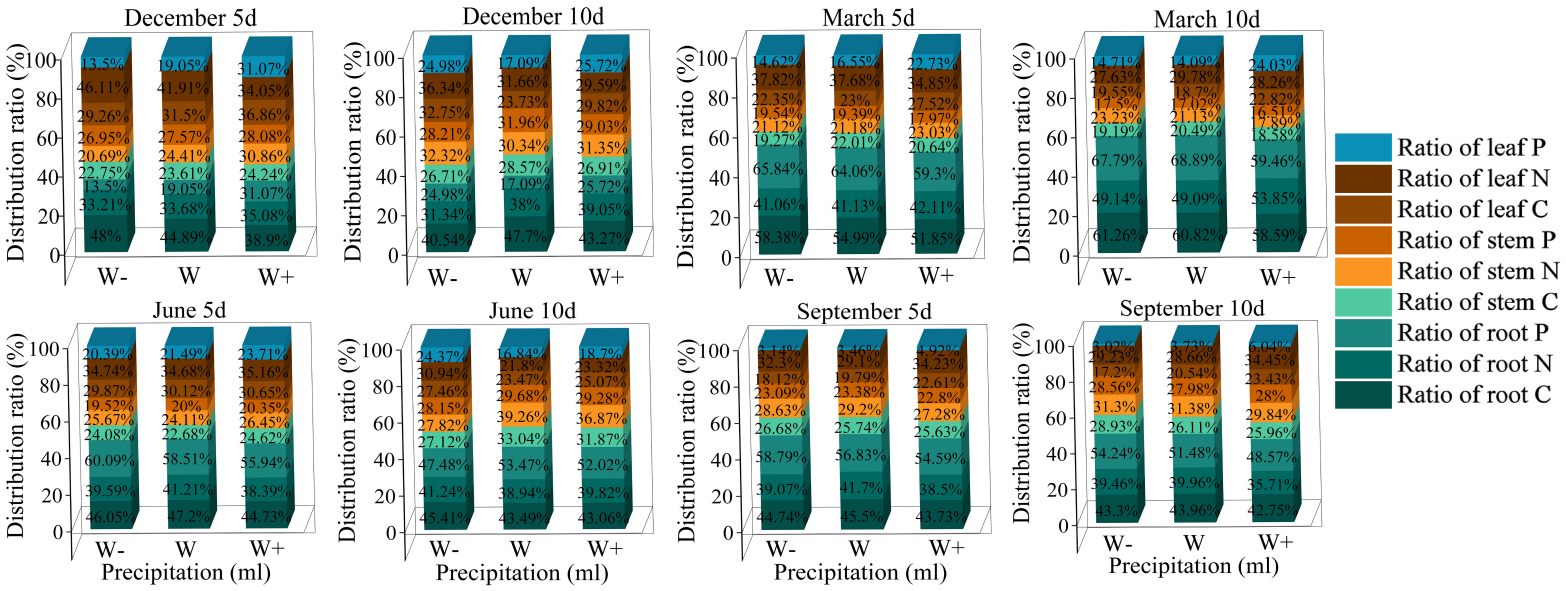
The Effect of Rainfall Patterns on the Distribution of Carbon, Nitrogen, and Phosphorus Nutrients in *Fraxinus malacophylla* Hemsl.

As shown in **Figure 5**, the allocation of C, N, and P varies among different organs. In the 5 days of rainfall treatment at the beginning of the dry season, except for the proportion of nutrient accumulation in leaves N, P, and roots C, the proportion of other nutrients increases with the increase of rainfall; In the 10 days of rainfall treatment, the proportion of root carbon was the highest under normal rainfall treatment, with a value of 47.70%. The proportion of roots N and P was the highest under increased rainfall treatment. The nutrient ratios of root N, stem C, P, and leaf C, N, P in *Fraxinus malacophylla* Hemsl seedlings increase with the increase of rainfall under different rainfall durations at the end of the dry season. During the rainy season, the overall proportion of nutrients in each organ shows that the 5-day rainfall duration is higher than the 10-day rainfall, indicating that high-frequency and short-term rainfall promotes nutrient accumulation of plants.

#### Nutrient accumulation and stoichiometric ratio per plant

**Figure 6:**
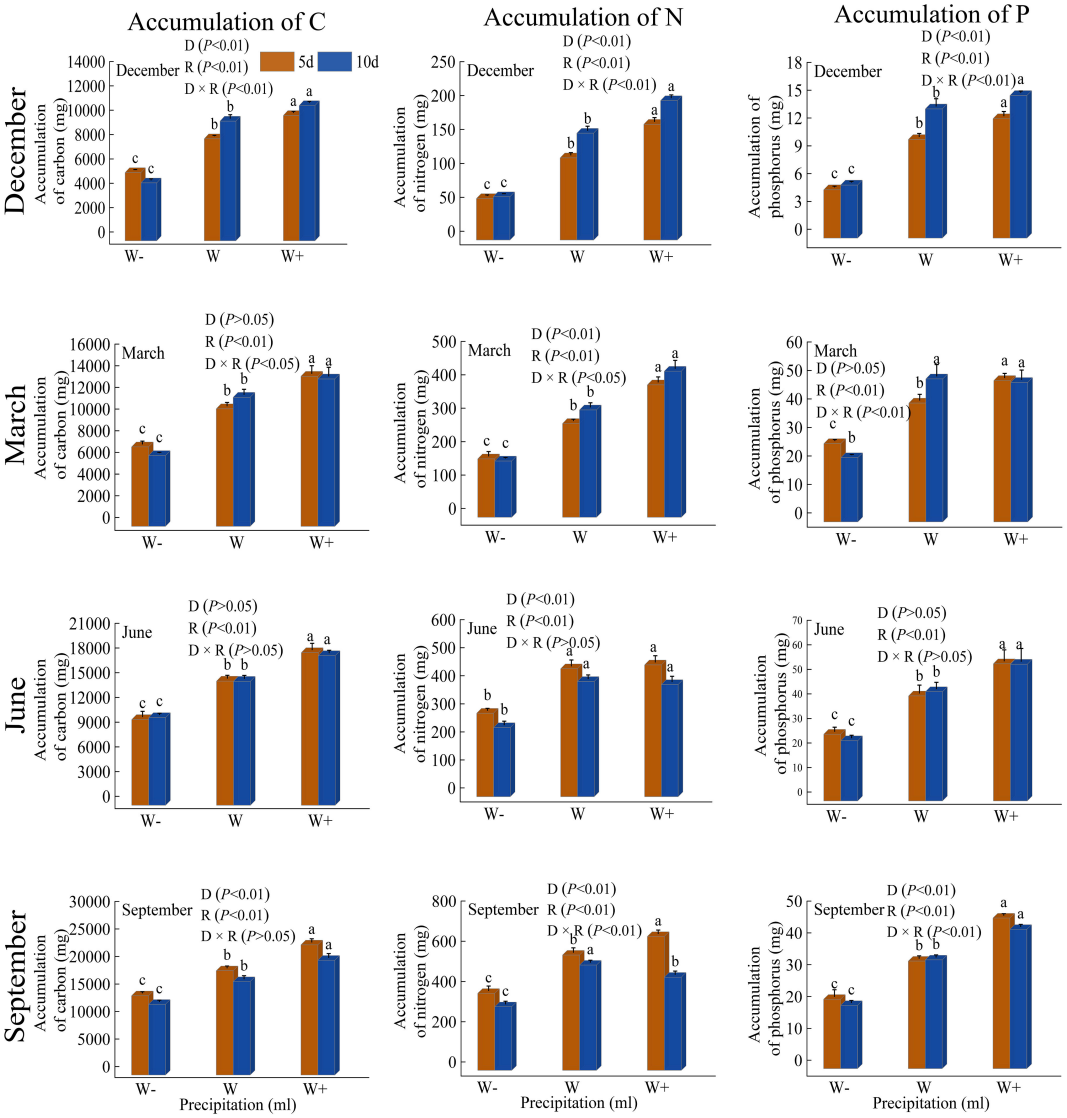
Impact of Rainfall Pattern on the Accumulation of C, N, and P in a Single Plant of *Fraxinus malacophylla* Hemsl.

There are certain differences in the accumulation of C, N, and P in individual seedlings of *Fraxinus malacophylla* Hemsl under different rainfall patterns (**Figure 6**). During the dry season period, nutrient accumulation was significantly different (*P*<0.05) under both rainfall duration and rainfall treatments, except for carbon accumulation in March, and the interaction between the two was also significantly different (*P*<0.05); With the increase in rainfall, the accumulation of C, N, and P nutrients showed an upward trend, indicating that increasing rainfall treatment can increase the nutrient content of plants, thereby promoting their growth and development. During the rainy season, the duration and impact of rainfall on the nutrient content of *Fraxinus malacophylla* Hemsl are not consistent; At the beginning of the rainy season, there was only a significant difference in N accumulation between rainfall duration and rainfall treatment, while at the end of the rainy season, there were significant differences in nutrient accumulation (*P*<0.01). In conclusion, with the increase in rainfall, the accumulation of nutrients per plant showed an upward trend, and the 10-day was higher than the 5-day rainfall in the dry season period, while the 5-day rainfall effect was better than the 10-day rainfall in the rainy season period.

**Figure 7:**
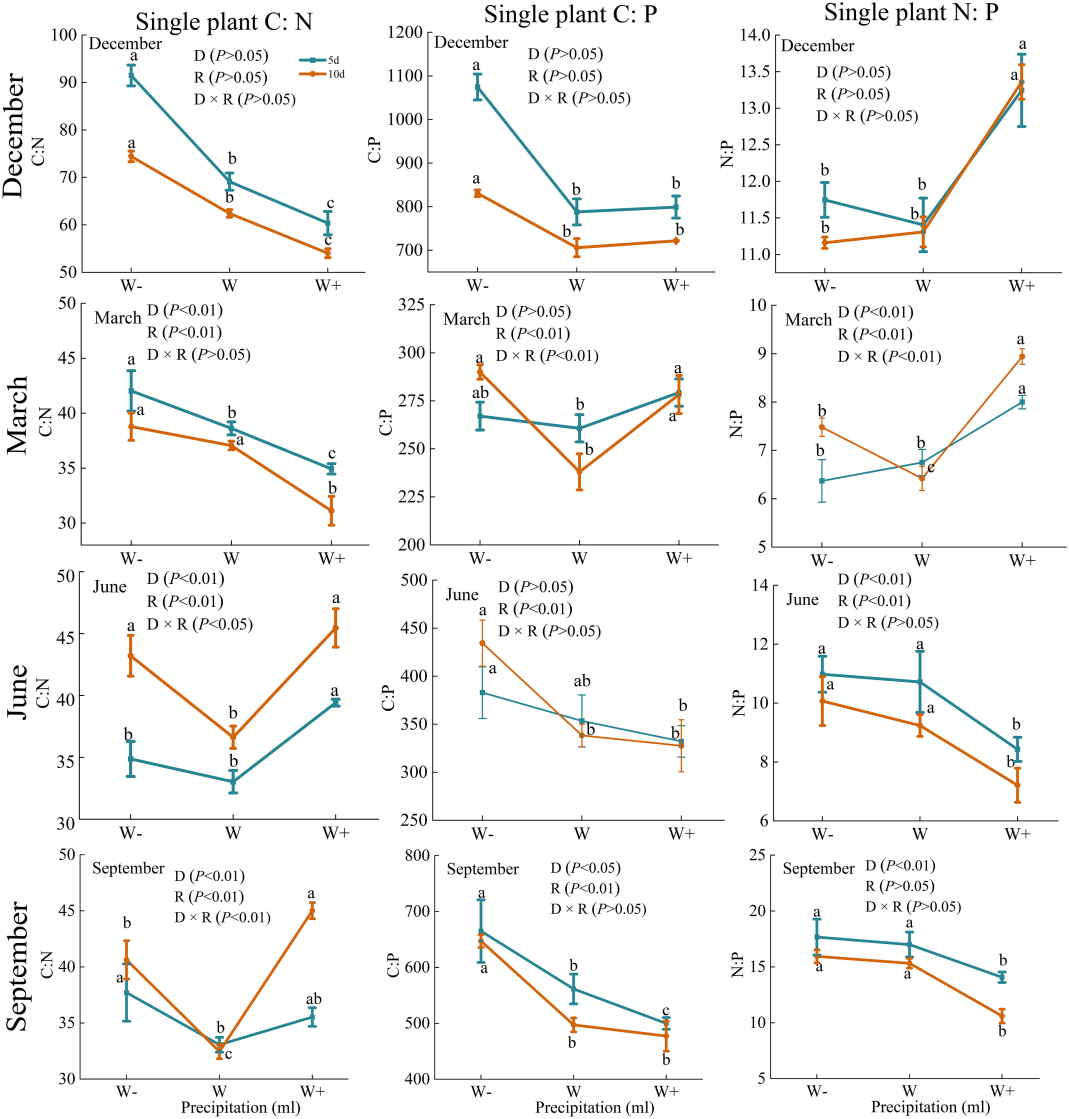
The impact of rainfall pattern on the stoichiometric ratios of C, N, and P for individual plants in *Fraxinus malacophylla* Hemsl.

As shown in **Figure 7**, the stoichiometric ratio of carbon, nitrogen, and phosphorus per plant varies under different rainfall patterns. At the beginning of the dry season, there were significant differences (*P*<0.01) in the C: N, C: P, and N: P values of *Fraxinus malacophylla* Hemsl under different rainfall durations and treatments, and the C: N and C: P values were both highest under reduced water treatment, while the N: P values were highest under increased rainfall treatment. At the end of the dry season, the C: P and N: P values showed a trend of first decreasing and then increasing with the increase of rainfall after 10 days of rainfall treatment, while C: N showed a downward trend. At the beginning of the rainy season, there were significant differences in C: N and N: P values under different rainfall durations and treatments (*P*<0.01). The C: N value showed a pattern of first decreasing and then increasing with the increase of rainfall, while C: P and N: P showed an overall decreasing trend. At the end of the rainy season, there was a significant difference (*P*<0.01) between C: N and C: P under the interaction of rainfall duration and rainfall, and the values of C: N and C: P showed a trend of first decreasing and then increasing with the increase of rainfall, while N: P showed no significant change between different rainfall levels.

### Content of NSC

**Figure 8:**
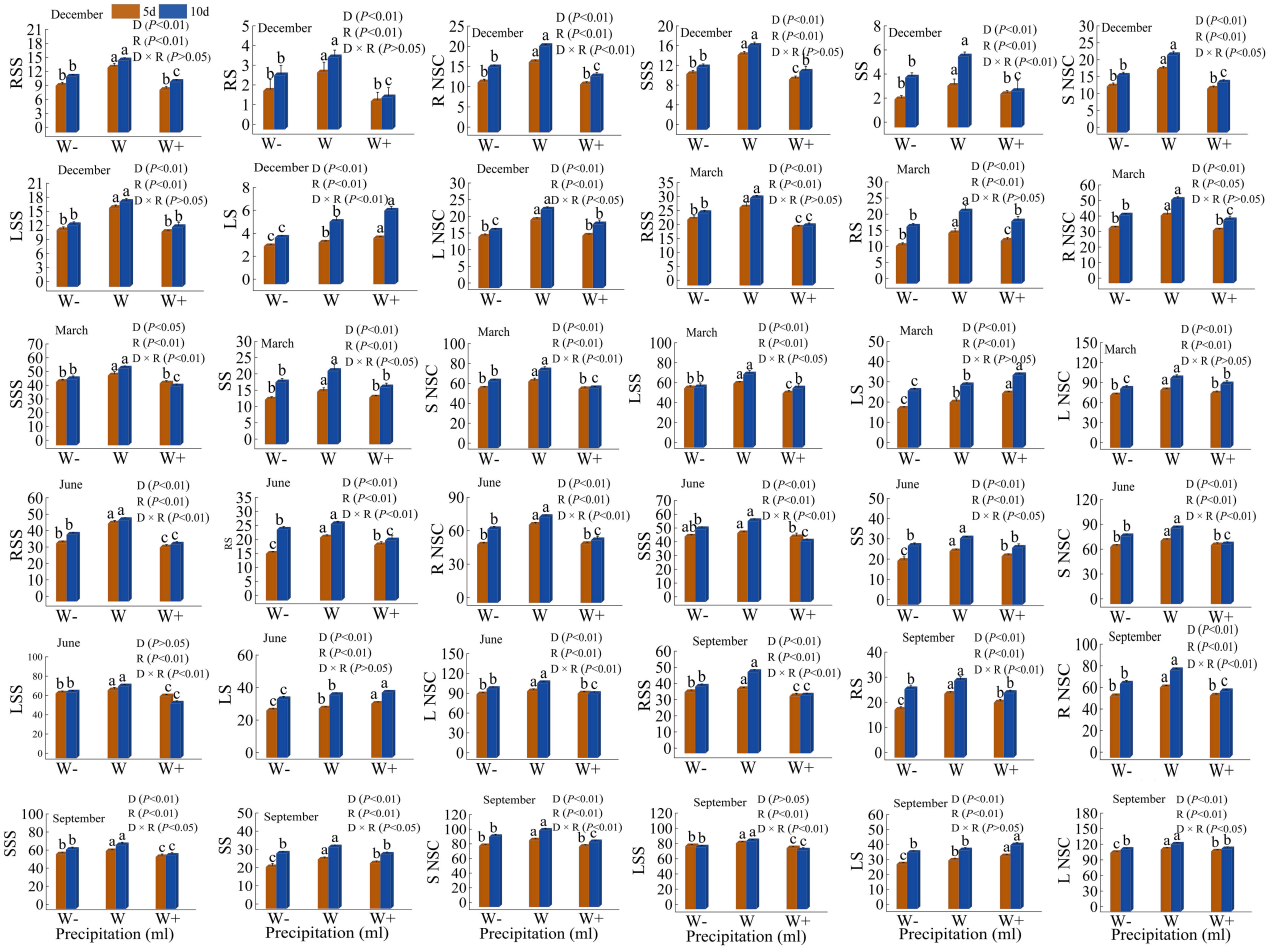
Effect of Rainfall Pattern on NSC Content in Roots, Stems, and Leaves of *Fraxinus malacophylla* Hemsl. RSS, RS, R NSC, SSS, SS, S NSC, LSS, LS, and L NSC in the figure represent the soluble sugar content (mg/g) of roots, starch content (mg/g) of roots, NSC content (mg/g) of roots, soluble sugar content (mg/g) of stems, starch content (mg/g) of stems, NSC content (mg/g) of stems, soluble sugar content (mg/g) of leaves, starch content (mg/g) of leaves, and NSC content (mg/g) of leaves. The same as below.

The soluble sugar and starch content in various organs of *Fraxinus malacophylla* Hemsl varies under different rainfall patterns (**Figure 8**). The non-structural carbohydrates of roots, stems, and leaves in *Fraxinus malacophylla* Hemsl were significantly different (*P*<0.01) under different rainfall duration and rainfall treatments, whether at the beginning or end of the dry season; While the significance was different under the interaction between the two. The content of soluble sugars and starch in each organ showed a pattern of increasing and then decreasing with the increase in rainfall (except for the content of starch in the leaves at the beginning and end of the dry season). In terms of root NSC content, it was 21.17 mg/g higher at the end of the dry season than at the beginning of the dry season under 5 days of normal rainfall, while it was 29.26 mg/g higher under 10 days of normal rainfall. In terms of stem NSC content, the increase in rainfall in the first 5 days of the dry season significantly decreased by 4.98mg/g compared to normal rainfall, and the increase in rainfall in the 10th day significantly decreased by 6.10mg/g. In terms of stem NSC content, the rain enhancement treatment was significantly reduced by 4.98 mg/g under 5 days rainfall duration and 6.10 mg/g under 10 days rainfall compared to normal rainfall at the beginning of the dry season. In terms of leaf NSC content, there was a significant difference (*P*<0.05) between normal rainfall and increased or decreased rainfall at the beginning or end of the dry season, while there was no difference (*P*>0.05) between increased and decreased rainfall. During the rainy season, there were significant differences (*P*<0.01) in non-structural carbohydrates (excluding soluble sugars in leaves) of various organs under different rainfall durations and rainfall, and the differences were different under the interaction of the two. Under a 5-day rainfall duration, the NSC content in the root of the *Fraxinus malacophylla* Hemsl under normal rainfall during the early rainy season is 1.33 and 1.34 times that of the increased and decreased rainfall, respectively, while at the end of the rainy season, it is 1.14 and 1.15 times that of the increased and decreased rainfall; At the beginning of the rainy season, the NSC content in the stems was 5.30 and 7.21mg/g higher in normal rainfall compared to increased and decreased rainfall, while 8.70 and 7.73mg/g higher at the end of the rainy season; At the beginning of the rainy season, the NSC content of leaves in normal rainfall increased by 3.41% and 4.76% compared to increased and decreased rainfall, respectively, while at the end of the rainy season, it increased by 3.04% and 5.50%. Under 10 days of rainfall duration, the increased and decreased rainfall of NSC content in the roots of *Fraxinus malacophylla* Hemsl at the beginning of the rainy season were 20.80 and 10.94mg/g lower than normal rainfall, and 19.41 and 12.42mg/g lower than normal rainfall at the end of the rainy season; The increased and decreased rainfall of NSC content in stems during the early rainy season was 27.17% and 11.69% lower than normal rainfall, respectively, and 17.75% and 8.99% lower than normal rainfall at the end of the rainy season; The increased and decreased rainfall treatment of NSC content of leaves at the beginning and at the end of the rainy season was significantly lower in the rain treatment than in the normal rainfall treatment (*P*<0.05).

In summary, the content of soluble sugar, starch, and NSC in the roots, stems, and leaves of *Fraxinus malacophylla* Hemsl seedlings showed a regulation of initial increase and subsequent decrease in different rainfall durations and treatments during the dry and rainy seasons. Moreover, 10 days of rainfall treatment promoted the accumulation of soluble sugar and starch content in various organs of *Fraxinus malacophylla* Hemsl.

### Relationship among nutrients of root, stem, leaf, and between NSC

#### Relationship between C, N, and P of root, stem, leaf

**Figure 9:**
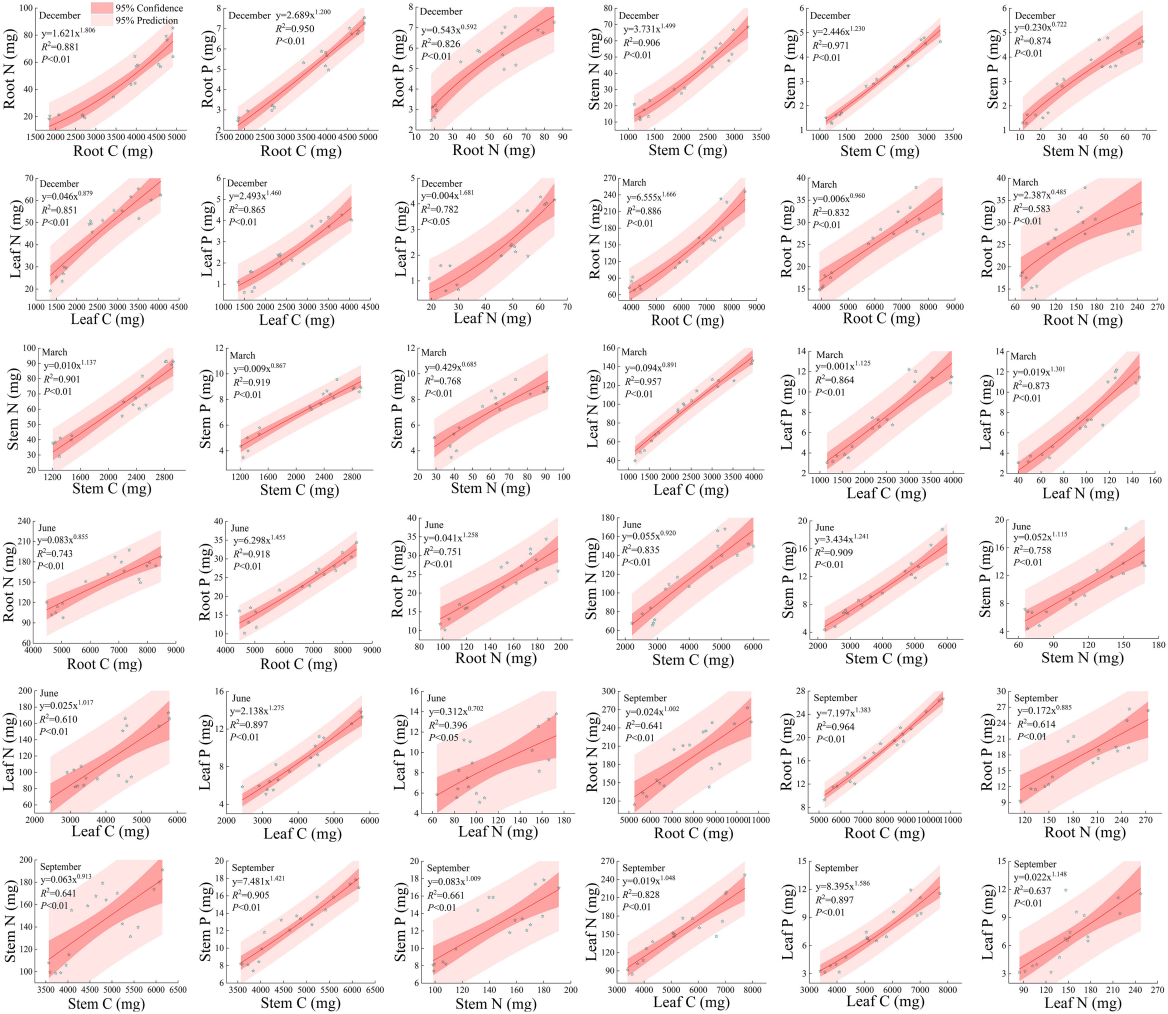
The Relationship between Carbon, Nitrogen, and Phosphorus in the Roots, Stems, Leaves of *Fraxinus malacophylla* Hemsl.

As shown in **Figure 9**, there is a significant positive correlation (*P*<0.05) between different elements in roots, stems, and leaves under different rainfall patterns during the dry and rainy seasons. Except for the root N and root P at the end of the dry season and the leaf N and leaf P at the beginning of the rainy season, the fitting degree *R* ^2^ of the non-linear curve-fitting between other elements is higher than 0.60, and the majority is above 0.75, with the highest fitting degree of 0.971. From this, it can be seen that there is a close correlation between the accumulation of C, N, and P in the roots, stems, and leaves, which also indicates that the nutrient elements C, N, and P are interdependent; It also indicates that the accumulation of one of the nutrients C, N, and P can infer the accumulation of the other two elements. From **Figure 9**, it can also be seen that during the dry season, the non-linear curve-fitting power function index between the nutrient elements in the roots, stems, and leaves of the *Fraxinus malacophylla* Hemsl fluctuates between 0.485 and 1.806. At the beginning of the dry season, except for the accumulation rates of P in the root, P in the stem, P and N in the leaves of *Fraxinus malacophylla* Hemsl, the nutrient accumulation rates in other organs are all greater than 1, indicating that with the increase of C or N element accumulation, the nutrient accumulation in other organs also increases. At the beginning of the dry season, except for the accumulation rate of P in the roots, stems, leaves, and N in the leaves of the *Fraxinus malacophylla* Hemsl the nutrient accumulation rates in other organs are greater than 1, indicating that with the increase of C or N element accumulation, the nutrient accumulation in other organs also increases. At the end of the dry season, from the perspective of nutrient accumulation rate, only root N, stem N, and leaf P had nutrient accumulation rates greater than 1, while the other nutrient accumulation rates were lower than 1. During the rainy season, the non-linear curve-fitting power function index of nutrient elements in various organs of *Fraxinus malacophylla* Hemsl is between 0.702 and 1.586. Specifically, at the beginning of the rainy season, except for the nutrient accumulation rates of root N, stem N, and leaf P that are less than 1, the accumulation rates of other nutrients are all higher than 1; But at the end of the rainy season, accumulation rate of only the root P was less than 1. In a word, there is a significant correlation between different elements in roots, stems, and leaves under different rainfall patterns, manifesting that the increase of one element in each organ also leads to the continuous accumulation of the other two elements.

#### Relationship between soluble sugars and starch in roots, stems, and leaves

**Figure 10:**
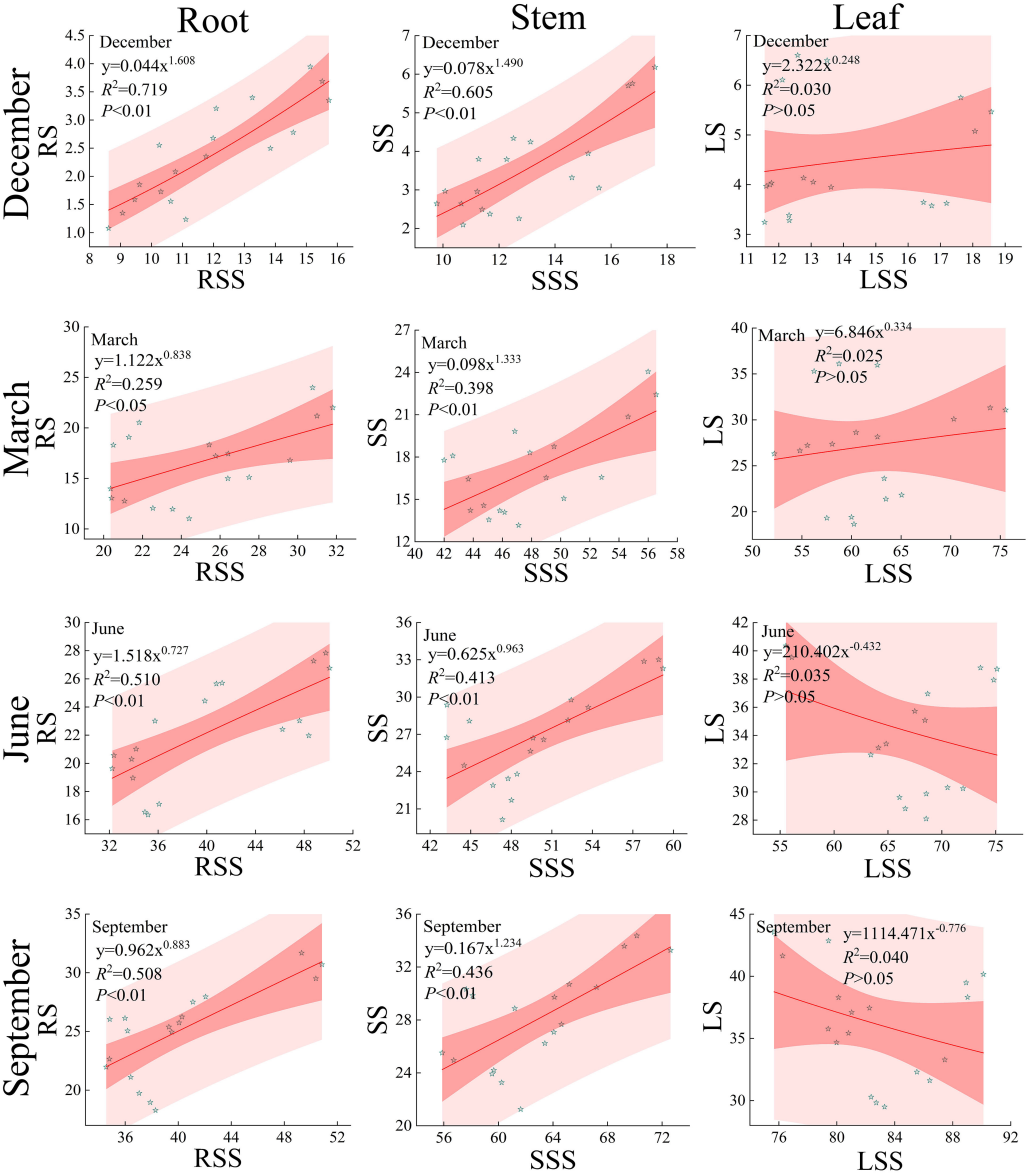
Relationship between soluble sugars and starch in the roots, stems, and leaves of the *Fraxinus malacophylla* Hemsl.

The correlation between soluble sugars and starch in the roots, stems, and leaves of *Fraxinus malacophylla* Hemsl varies under different rainfall patterns (**Figure 10**). During the dry and rainy season, except for the insignificant correlation between soluble sugar and starch content in the leaves of the *Fraxinus malacophylla* Hemsl (*P*>0.05), there was a highly significant positive correlation between soluble sugar and starch in the roots and stems (*P*<0.05). During the dry season, the non-linear curve-fitting degree *R* ^2^ between soluble sugars and starch in roots, stems, and leaves were 0.719, 0.605, 0.030, 0.259, 0.398, and 0.025, respectively, and there was a significant positive correlation between soluble sugars and starch in roots and stems (*P*<0.01); This indicates that the soluble sugar and starch content in the roots and stems of the *Fraxinus malacophylla* Hemsl are closely related, and the starch content continues to increase with the increase of soluble sugar. During the rainy season, the power function fitting indices of the non-linear curve between soluble sugar and starch in leaves are -0.432 and -0.776, which are negatively correlated. This indicates that the starch content decreases with the increase of soluble sugar content, and also indicates that the starch content continuously transforms into soluble sugar. However, the fitting index of soluble sugar and starch in roots and stems is both greater than 0, indicating a positive correlation between soluble sugar and starch.

In conclusion, under different rainfall patterns, there is both a positive and a negative correlation between the soluble sugar and starch content in various organs of *Fraxinus malacophylla* Hemsl. During the dry season, the starch content of each organ increases with the increase of soluble sugar. During the rainy season, with the increase of soluble sugar, the starch content in the roots and stems gradually increases and soluble sugar continuously transforms into starch. However, in the leaves, with the increase of soluble sugar, the starch content decreases, and starch transforms into soluble sugar.

#### Allometric growth relationship between roots, stems, and leaves

Under different rainfall durations during the dry and rainy seasons, there was a significant correlation (*P*<0.01) between the biomass allometric growth relationships among various organs of the *Fraxinus malacophylla* Hemsl (**Table 5**). At the beginning of the dry season, there were significant differences in biomass between stem-root and leaf-root, except for the leaf-stem biomass that did not show any difference between different rainfall durations; And the growth slope of seedlings with a 5-day rainfall duration is significantly greater than 1.0, indicating an allometric growth relationship, while under a 10-day rainfall duration, it is an isokinetic growth relationship, evidencing that they have a common slope of 0.976 and 0.641, respectively. However, the allometric growth relationship between leaves and stems is an isokinetic under different rainfall durations. At the end of the dry season, the allometric growth relationship of stem-root biomass showed under different rainfall durations, indicating that different rainfall durations were beneficial for the accumulation of root biomass in *Fraxinus malacophylla* Hemsl seedlings; During 5 days of rainfall duration, the relationship between the biomass of leaves-stems showed an isokinetic growth, indicating that the accumulation of leaf biomass was greater than that of stems. At the beginning of the rainy season, except for the significant difference in slope between the stem root and leaf stem biomass under 10-day rainfall duration treatment and *P_-1.0_* (*P*<0.05), there was no significant difference in other treatments (*P*>0.05). But, at the end of the rainy season, the biomass of the stem-root and leaf-root showed an isokinetic growth relationship under different rainfall durations, while the leaf-stem showed an allokinetic growth relationship under 10 days of rainfall duration.

**Table 5:** Analysis of allometric growth of biomass in various organs of *Fraxinus malacophylla* hemsl seedlings under rainfall patterns.

Month	Trait	Precipitation interval	*R* ^2^	*P*	Slope	95%confidence interval		*F* value	*P*_-1.0_	Type
December	Stem(y)-Root(x)	5d	0.896	0.000	1.880a	1.415	2.498	30.559	0.001	*A*
		10d	0.955	0.000	0.967b	0.801	1.169	0.170	0.693	*I*
	Leaf(y)-Root(x)	5d	0.846	0.000	1.863a	1.321	2.626	20.017	0.003	*A*
		10d	0.568	0.019	0.641b	0.367	1.120	3.417	0.107	*I*
	Leaf(y)-Stemt(x)	5d	0.910	0.000	0.991a	0.760	1.292	0.007	0.938	*I*
		10d	0.552	0.022	0.663a	0.376	1.169	2.796	0.138	*I*
March	Stem(y)-Root(x)	5d	0.973	0.000	1.279a	1.103	1.482	15.717	0.005	*A*
		10d	0.913	0.000	0.733b	0.565	0.953	7.942	0.026	*A*
	Leaf(y)-Root(x)	5d	0.965	0.000	1.325a	1.122	1.565	16.352	0.005	*A*
		10d	0.911	0.000	1.160a	0.891	1.509	1.735	0.229	*I*
	Leaf(y)-Stemt(x)	5d	0.923	0.000	1.036b	0.811	1.324	0.117	0.742	*I*
		10d	0.867	0.000	1.581a	1.148	2.178	11.851	0.011	*A*
June	Stem(y)-Root(x)	5d	0.808	0.001	1.270b	0.867	1.862	2.126	0.188	*I*
		10d	0.652	0.009	2.182a	1.316	3.618	14.917	0.006	*A*
	Leaf(y)-Root(x)	5d	0.930	0.000	1.140a	0.902	1.441	1.736	0.229	*I*
		10d	0.451	0.048	1.097a	0.589	2.043	0.110	0.750	*I*
	Leaf(y)-Stemt(x)	5d	0.919	0.000	0.898a	0.698	1.155	1.007	0.349	*I*
		10d	0.509	0.031	0.503a	0.278	0.908	7.870	0.026	*A*

P_-1.0_ in the table represents the significant difference between the slope and the theoretical value of 1.0, different lowercase letters indicate significant differences (P<0.05) between the different rainfall duration for biomass of each organ of Fraxinus malacophylla Hemsl seedlings. A: allometric relationship, I: isometric relationship. R^2^ is the coefficient of determination, and P is the significance.

### Principal component analysis

**Figure 11:**
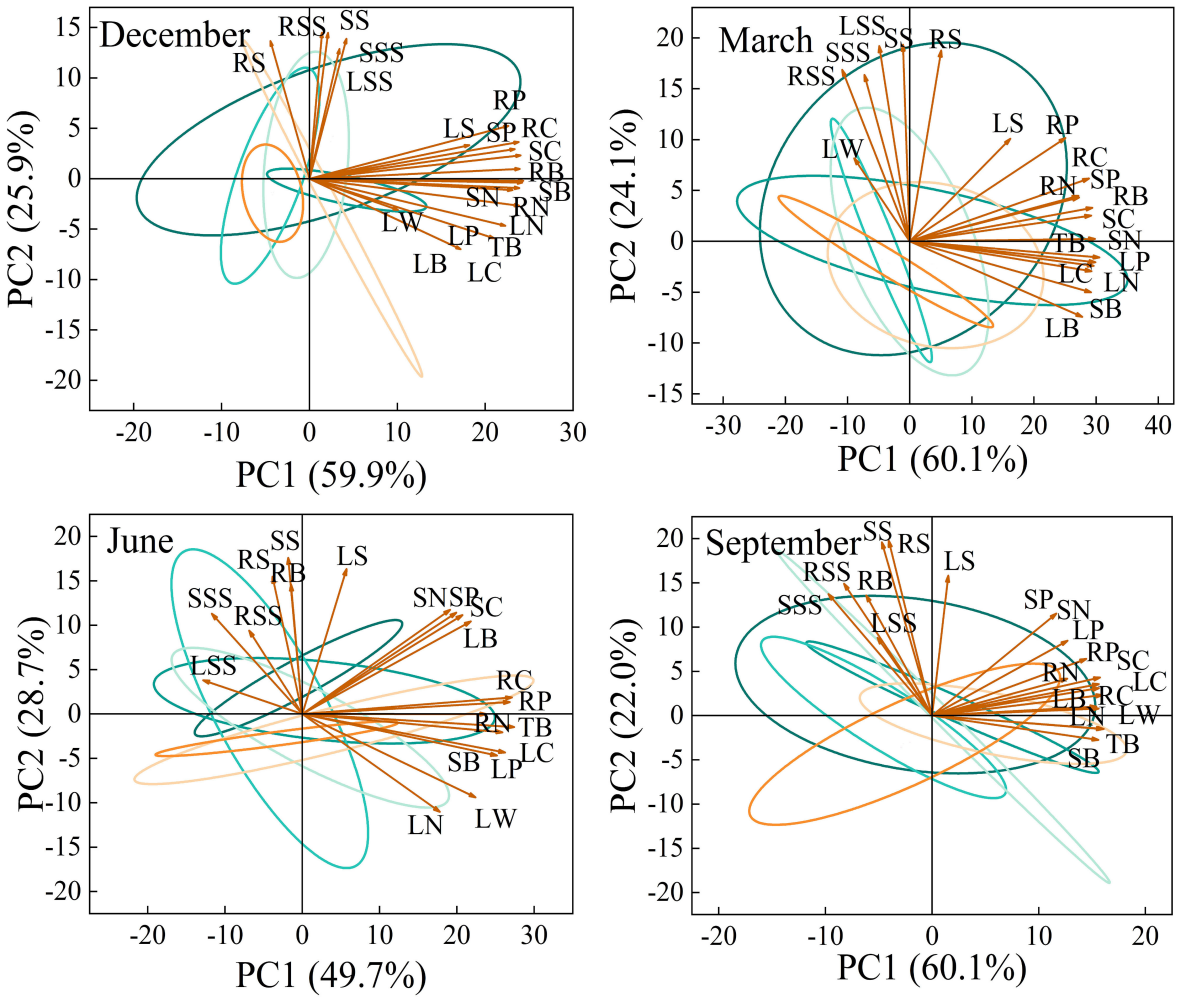
Principal Component Relationship of Root, Stem, and Leaf Biomass, Nutrients, and NSC Content of *Fraxinus malacophylla* Hemsl under Rainfall Patterns.

The main purpose of principal component analysis is to use fewer variables to explain the degree of variation of various indicators, in order to comprehensively and systematically analyze the impact of changes in rainfall patterns on the growth of *Fraxinus malacophylla* Hemsl seedlings. As shown in **Figure 11**, the principal component relationships of biomass, nutrient accumulation, and NSC content in various organs of *Fraxinus malacophylla* Hemsl vary under different rainfall patterns during the dry and rainy seasons. At the beginning of the dry season, two principal components (PC1 and PC2) were extracted from the measured indicators, with a cumulative variance contribution rate of 85.8%, representing most of the information of each indicator. The contribution rate of PC1 is 59.9%, and RB, SB, TB, RC, SC, RN, SN, LN, RP, SP, and LP have the larger forward load traits; The variance interpretation rate of PC2 is 25.9%, which includes the larger forward load values for RSS, SSS, LSS, RS, and SS. At the end of the dry season, the variance contribution rate of PC1 is 60.1%, LW, RSS, SSS, and SS have the larger negative load values, the contribution rate of PC2 is 24.1%, SB, LB, TB, LC, LN, and LP have the larger negative load values, and the cumulative variance contribution rate is 84.2%. At the beginning of the rainy season, with a cumulative variance contribution rate of 78.4%, the contribution rate of PC1 was 49.7%, and the contribution rate of PC2 was 28.7%; At the end of the rainy season, the cumulative contribution rate of variance was 82.1%, the contribution rate of PC1 was 60.1%, and the contribution rate of PC2 was 22.0%. From this, it can be seen that under different rainfall patterns, the principal component analysis of each indicator shows that the cumulative contribution rate of variance during the dry and rainy seasons is higher than 75%, which can represent the vast majority of information.

**Figure 12:**
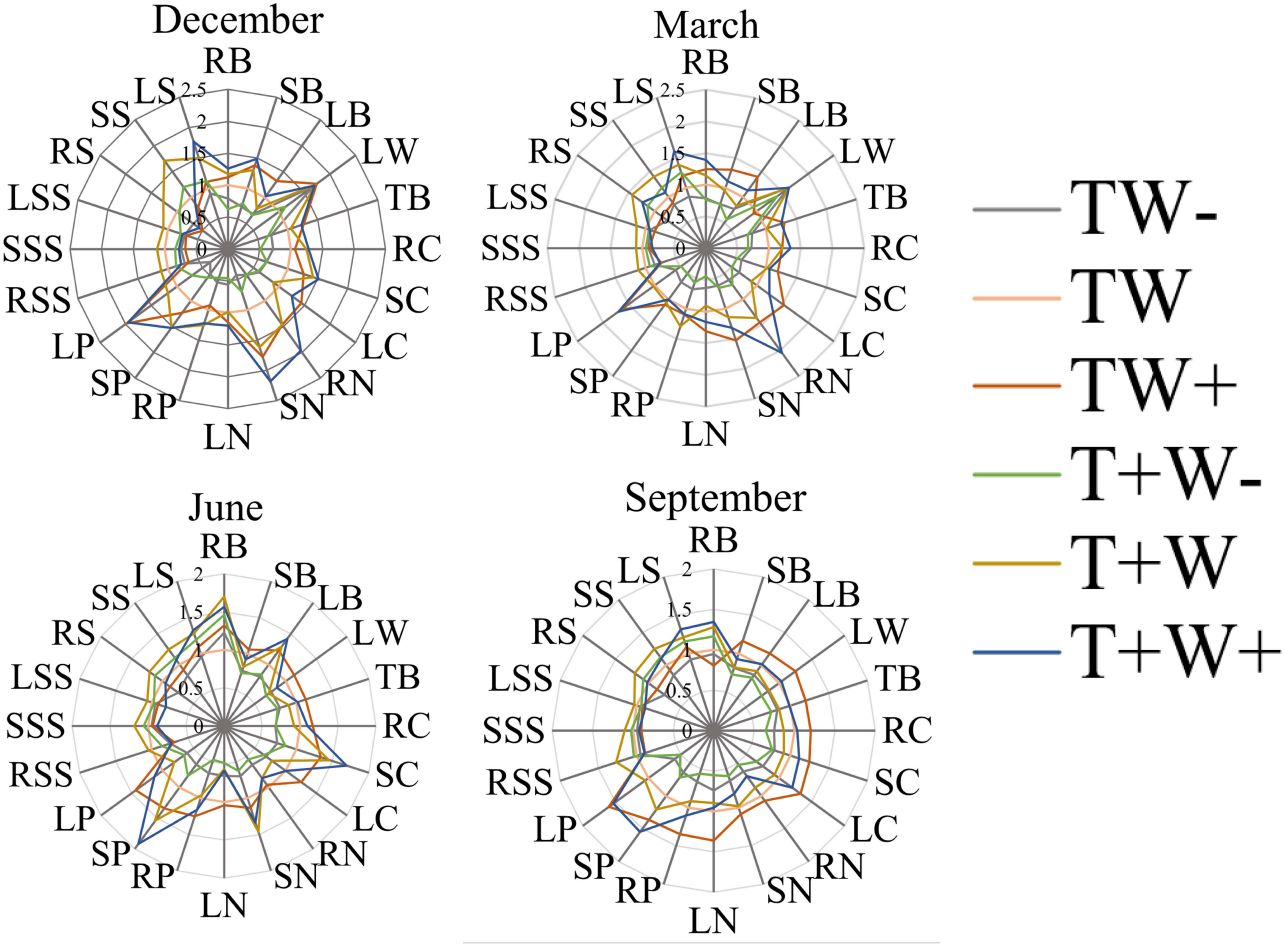
Effects of Rainfall Patterns on the Biomass, Nutrients, and NSC Content Changes of the Roots, Stems, and Leaves of *Fraxinus malacophylla* Hemsl.

As shown in **Figure 12**, the content changes of each indicator show different trends under different rainfall patterns. During the dry season, LP was the highest under TW_+_ and T_+_W_+_ treatments, with a value of 2.0; TB, RC, SC, LC, RN, SN, LN, RP, SP, and LP increased with the increase of rainfall under the same rainfall duration; LW, RB, RP, RC, and TB have the lowest rainfall under TW - treatment, with a rain reduction treatment in the 5 days, while the majority of indicators have the best effect under TW_+_ treatment. During the rainy season, SP shows a peak under T_+_W_+_ treatment, while LW, TB, LC, RN, SN, and LN are best treated with 5 days of increased rainfall under TW_+_ treatment, indicating that increasing rainfall on *Fraxinus malacophylla* Hemsl during normal rainfall hours is beneficial for plant growth.

## Discussion

### Biomass accumulation and allocation of root, stem, and leaf

The occurrence of extreme rainfall events (prolonged rainfall duration and heavy rainfall) is caused by global climate change (Diffenbaugh and Giorgi, 2012). Plant biomass is affected by water mainly (Yu et al., 2011). Studies have shown that increasing rainfall promotes the accumulation of plant biomass while prolonging rainfall duration has the opposite effect (Fay et al., 2000; Ansley et al., 2014). However, most studies only focus on grassland ecosystems, and few have studied the response of vegetation in rocky desertification areas to climate change. In this study, the accumulation of root and stem biomass in the early dry season was higher in the 10-day rainfall treatment than in the 5-day rainfall treatment, and this phenomenon only appeared in the root biomass at the end of the dry season; During the rainy season, the biomass accumulation of roots, stems, and leaves showed that 5-day rainfall was better than 10-day rainfall. Overall, low-frequency heavy rainfall (10 days of rainfall) reduced the accumulation of biomass in the *Fraxinus malacophylla* Hemsl; Moreover, there were significant differences in the biomass accumulation of various organs under different rainfall treatments, similar to previous research results (Holub, 2002; Ansley et al., 2014; Zhang J. et al., 2020). Usually, plant biomass increases with increasing rainfall in areas with water scarcity, as water scarcity is a limiting factor in these environments (Weiss et al., 2004; Kardol et al., 2010).

Biomass is an important indicator of plant energy accumulation, and the distribution differences of biomass in various organs can reflect the growth strategy of plants (Westoby et al., 2002). However, with the increase in rainfall, the soil moisture content increases, leading to differences in the absorption and utilization of soil moisture by plants, which in turn affects the growth of plant roots, stems, and leaves and the allocation of biomass. Numerous studies have shown a positive correlation between the accumulation of plant biomassand annual rainfall (Knapp and Smith, 2001; Bai et al., 2008). Increasing rainfall significantly promotes the growth of the aboveground part of Quercus mongolica seedlings, while reducing drought caused by rainfall inhibits both aboveground and underground biomass, thereby reducing the regeneration potential of Quercus mongolica seedlings (Dong and Sang, 2012). Yet extending the rainfall interval will promote the accumulation of aboveground, underground, and total biomass in plants (Swemmer et al., 2007; Heisler-white et al., 2009), but research results in the wetter grasslands of North America have shown the opposite (Fay et al., 2000; Knapp et al., 2002). In this study, at the beginning of the dry season, extending the duration of rainfall can promote the allocation of root and stem biomass. It may be that extending the duration of rainfall increases the duration of plant drought stress tolerance to obtain more water sources, the seedlings of *Fraxinus malacophylla* Hemsl expand their root system distribution by increasing the accumulation of underground biomass, thereby enhancing their drought resistance ability; It is basically consistent with previous research results (Knapp et al., 2002). During the rainy season, the same amount of rainfall prolongs the duration of rainfall and has an inhibitory effect on the distribution of biomass in the roots, stems, and leaves of the *Fraxinus malacophylla* Hemsl; Under the same rainfall duration, the biomass of each organ increases with the increase of rainfall, which is consistent with previous studies on the response of most shrubs in desert ecosystems to changes in precipitation (Bai et al., 2008). In this study, the allocation of biomass in the roots, stems and leaves of the *Fraxinus malacophylla* Hemsl showed that roots>leaves>stems, and plants have growth strategies. However, according to the optimal partitioning theory, in order to reduce water constraints, plants prioritize the growth of underground roots to promote water absorption, thus typically increasing root biomass (Hertel et al., 2013), resulting in the highest accumulation of biomass in the roots of the *Fraxinus malacophylla* Hemsl under different rainfall patterns. However, according to the optimal allocation theory, in order to reduce water constraints, plants prioritize the growth of underground roots to promote water absorption, thereby promoting an increase in root biomass (Hertel et al., 2013). Therefore, under different rainfall patterns, the accumulation of root biomass in the *Fraxinus malacophylla* Hemsl is the highest.

### Accumulation and allocation of nutrients in roots, stems, and leaves

Water changes can affect the absorption, transport, allocation, and storage of organic carbon, total nitrogen, and total phosphorus in plants, thereby altering the accumulation and distribution of nutrients in plant roots, stems, and leaves (Rouphael et al., 2012). The accumulation of C, N, and P nutrients in plant roots, stems, and leaves is one of the important indicators reflecting the nutritional status of plants, which can reflect the growth status of plants in adverse environments (Shi et al., 2021). In this study, during the dry and rainy seasons, the accumulation of C, N, and P in the roots, stems, and leaves of *Fraxinus malacophylla* Hemsl significantly increased with the increase in rainfall. Moreover, during the rainy season, the duration and increase of rainfall have a certain promoting effect on the accumulation of nutrients in the *Fraxinus malacophylla* Hemsl, as reported by Gao et al. (2014). This is because the release and migration of nutrients in the soil, as well as the absorption of nutrients by plants, are closely related to soil moisture. The increase in rainfall leads to an increase in soil moisture content, which accelerates the release and migration rate of soil nutrients, and further enhances the absorption and utilization of soil nutrients by plants; Therefore, under high-frequency rainfall increase treatment during the rainy season, the roots, stems, and leaves of *Fraxinus malacophylla* Hemsl seedlings have higher nutrient accumulation. This study found that under different rainfall treatments, the nutrient allocation of various organs in the *Fraxinus malacophylla* Hemsl showed the highest carbon, followed by nitrogen, and the lowest phosphorus, which is the same as previous studies (Ye et al., 2014). The highest carbon accumulation may be due to the “carbon fixation” function of plants, which means that plants convert CO_2_ in the air into organic matter through photosynthesis and store it in the body, thereby providing energy for themselves; Therefore, the carbon accumulation in each organ of the *Fraxinus malacophylla* Hemsl is the highest. Secondly, in this study, the accumulation of carbon, nitrogen, and phosphorus nutrients among roots, stems, and leaves showed that root>leaf>stem; There is a certain trade-off relationship between the stoichiometric characteristics of the various organs of the *Fraxinus malacophylla* Hemsl, which reflects the regulatory strategies of plants in acquiring resources and allocating nutrients under different rainfall treatments. The nutrient content is the best among the roots of *Fraxinus malacophylla* Hemsl, indicating that the roots of *Fraxinus malacophylla* Hemsl enhance their uptake of water and nutrients, thereby transporting them to the leaves through the stem and increasing the photosynthetic rate of plants; At the same time, the nutrient content (C, N, P) in the leaves can better maintain plant photosynthesis, thereby protecting the material and energy sources for the growth of plant roots, stems, and leaves, indicating that there is a certain survival strategy between the roots, stems, and leaves of the *Fraxinus malacophylla* Hemsl. In addition, this study indicates that during the dry and rainy season, the nutrient ratio of each organ of *Fraxinus malacophylla* Hemsl seedlings as a whole showed that roots>leaves>stems, and the nutrient content tended to be accumulated in the roots; The preliminary analysis was that when there was a lack of water, the plant allocated more nutrients to their roots in order to survive, which led to the rapid growth and development of the root system, allowing the roots to grow and develop rapidly, thereby obtaining deeper and more nutrient content.

The stoichiometries value of C, N, and P are important indicators determining plant nutrient limitations (Wang et al., 2013; Mao et al., 2016). The N and P contents in plant leaves can determine the limitations of plant nutrients in this environment (Zhang et al., 2019). When N: P<14, plant growth is mainly limited by N elements. When 14<N: P<16, plant growth is limited by N and P elements. When N: P>16, plant growth is limited by P elements. In this study, regardless of the dry season or rainy season, the N: P values of the leaves of the *Fraxinus malacophylla* Hemsl were greater than 16 under different rainfall treatments (**Figure 4**), and the growth of the *Fraxinus malacophylla* Hemsl was mainly limited by P, consistent with the findings of Han et al. (2011); Han et al. (2011) also believed that the nutrient element limiting plant growth on land in China is mainly P, and prolonged rainfall and increased rainfall limit plants to P, which may be related to the availability of nutrient elements in the soil. This is due to low-frequency heavy rainfall, soil nutrients are flushed away, and the resources available to the plants through the soil will be reduced.

### NSC content in roots, stems, and leaves

Plant NSC is a major intermediate product between photosynthesis (carbon uptake) and respiration (carbon consumption), and its storage size reflects the balance between carbon uptake and consumption (Poorter and Kitajima, 2007). Water is the main limiting factor for plant growth and development. Plants can regulate their metabolic processes through a series of physiological and biochemical reactions in water-deficient environments, thereby adapting to adverse environments and maintaining normal growth and physiological metabolism (Rosas et al., 2013). In water-deficient environments, the storage and transformation mechanism of plant NSC, such as the composition ratio and dynamic changes of soluble sugars and starch, is beneficial for maintaining the main functions of plants (growth, respiration, reproduction, etc.) (Thalmann and Santelia, 2017). In this study, the response pattern of NSC content in various organs of *Fraxinus malacophylla* Hemsl seedlings to changes in rainfall was different under different rainfall durations and rainfall treatments (**Figure 8**), and there were significant differences in different organs (*P*<0.05). Under the same rainfall duration, the soluble sugars and starch of each organ showed a pattern of change of first increasing and then decreasing with the increase of rainfall, which was the same as the results of the previous study (Wang et al., 2017). Under the same rainfall, the NSC content during extended rainfall duration is better than that during normal rainfall duration; And the soluble content is higher than the starch content, indicating that when plants are subjected to certain stress, the starch content decreases and the soluble sugar content increases to maintain cellular osmotic changes (Dietze et al., 2014; Hartmann and Trumbore, 2016). We also found a significant feature that both increasing and decreasing rainfall treatments have inhibitory effects on soluble sugar and starch content, as water deficiency can lead to a decrease in the turgor pressures of cambium cells, thereby inhibiting plant growth (Peters et al., 2020). Moreover, water deficiency can lead to weakened photosynthesis in plants, reduced synthesis of organic matter, and a decrease in soluble sugar and starch content (Körner, 2003). The increase in rainfall results in relatively good plant growth and higher nutrient accumulation in various organs (**Figure 3**), while the NSC content decreases. This indicates that plants consume NSC content to promote plant growth to increase nutrient accumulation and root respiration, as NSC content is related to root respiration and can increase energy for the emergence of new roots and nutrient absorption (Zhu et al., 2021). The soluble sugar and starch content during the rainy season is higher than that in the dry season, as the plant growth during the rainy season is higher than that during the dry season (Gheyret et al., 2021). This research result is also reflected in the biomass and nutrient accumulation status of the *Fraxinus malacophylla* Hemsl in this article.

#### Relationship among nutrients in roots, stems, and leaves

Carbon, nitrogen, and phosphorus are essential elements in plants, playing different ecological roles in the natural environment, and there is also a close correlation between them; Moreover, the nitrogen and phosphorus contents of plants have a certain effect on the production capacity and carbon fixation of terrestrial ecosystems (Tang et al., 2018). This is because nitrogen can promote the absorption of radioactive carbon in plants and promote the metabolic activity of plant growth (Kopittke et al., 2016); The interrelationship between nitrogen and phosphorus is very close (Zhao et al., 2023), and the N: P value of plant leaves can be used to determine the nutrient constraints on their growth and development (Wang et al., 2013; Mao et al., 2016). This study found that under different rainfall patterns, there was a significant positive correlation between carbon-nitrogen, carbon phosphorus, and nitrogen phosphorus in the same organ (*P*<0.05), and the majority of the curve fit *R* ^2^ was above 0.75, with the highest fit reaching 0.971 (**Figure 9**). It indicates that as the carbon content increases, the nitrogen and phosphorus content also increases; The reason is that plants fix carbon through photosynthesis, continuously forming dry matter, providing substrates and energy for various physiological and ecological processes, thus increasing the accumulation of nitrogen and phosphorus of the *Fraxinus malacophylla* Hemsl. N and P are essential mineral elements for the composition of cellular structural substances. The nutrient characteristics of N and P in different rainfall patterns not only reflect the growth characteristics of plants themselves but also the result of long-term adaptation to habitats. With the continuous change of rainfall, N and P elements show a significant positive correlation and synergistic change, indicating a certain interaction between N and P metabolism (Raven, 2015); It also indicates that the supply of P promotes the absorption and utilization of nitrogen by plants, and the supply of nitrogen also promotes the absorption and utilization of phosphorus by plants.

The accumulation of soluble sugars helps regulate osmotic stress in plant cells and promotes the protection of biomolecules and membranes (Irannejad and Shahbazian, 2004). The increase in soluble sugar content in plants can enhance cellular carbohydrate metabolism, maintaining it at optimal levels (Gibson, 2005). The reduction of starch can decompose into smaller molecules to increase the accumulation of soluble sugars in plant cells, leading to interconversions between the two (Ashraf and Harris, 2004). In this study, under different rainfall patterns, with the increase of soluble sugars in various organs of the *Fraxinus malacophylla* Hemsl, the starch content also increased, and soluble sugars continuously transformed into starch; During the rainy season, the leaves of the *Fraxinus malacophylla* Hemsl exhibited a continuous transformation of starch into soluble sugars (**Figure 10**), which is consistent with previous studies (Ashraf and Harris, 2004).

## Conclusion

In summary, the impact of different rainfall patterns during the dry and rainy seasons on the biomass, ecological stoichiometry, and NSC of *Fraxinus malacophylla* Hemsl seedlings. The results showed that the biomass of roots, stems, and leaves, as well as the accumulation of C, N, and P, increased with the increase in rainfall. The biomass and nutrient accumulation of each organ showed root>leaf>stem. And N: P>16, plant growth is limited by the P element. In the future, attention should be paid to the addition of P fertilizer in the management of *Fraxinus malacophylla* Hemsl. Except for the beginning of the dry season, prolonging the duration of rainfall in other periods inhibits the biomass accumulation of *Fraxinus malacophylla* Hemsl seedlings, and promotes the accumulation of C, N, and P nutrients and an increase in soluble sugar and starch content. The soluble sugar and starch content in various organs showed a pattern of first increasing and then decreasing with the increase of rainfall, and prolonging the rainfall interval significantly promoted the accumulation of NSC content. Reducing rainfall has an inhibitory effect on the biomass, nutrient accumulation, and NSC content of *Fraxinus malacophylla* Hemsl. The nutrient content of C, N, and P in each organ, as well as the content of soluble sugar and starch, showed extremely significant differences (*P*<0.05). The principal component analysis of each indicator shows that the cumulative variance contribution rate during the dry and rainy seasons is higher than 75%. This study explores the growth status of *Fraxinus malacophylla* Hemsl seedlings under the conditions of climate and environmental changes from the perspective of physiological growth, providing a theoretical basis for the cultivation, management, application, and popularization of *Fraxinus malacophylla* Hemsl in rocky desertification and karst areas.

In this study, we explored the response of biomass, NSCs, and carbon, nitrogen, and phosphorus to changes in rainfall patterns of indoor-cultivated *Fraxinus malacophylla* Hemsl plants. The significant changes in carbon, nitrogen, and phosphorus concentrations in various organs of the *Fraxinus malacophylla* Hemsl under changes in rainfall patterns are due to changes in nutrient absorption and distribution caused by changes in rainfall. This underscores the pivotal role of rainfall in shaping plant development, functionality, and metabolic processes, aligning with prior studies. Our findings emphasize the need for precise precipitation management strategies in *Fraxinus malacophylla* Hemsl cultivation. Our research resonates beyond the scientific realm, offering actionable guidance for cultivators striving to maximize the benefits of *Fraxinus malacophylla* Hemsl. While our study has provided valuable insights into indoor planting of *Fraxinus malacophylla* Hemsl, it is essential to acknowledge that further investigations should specifically delve deeper into the impact of enhanced rainfall during rainfall.

## Data availability statement

The original contributions presented in the study are included in the article/supplementary material. Further inquiries can be directed to the corresponding author.

## Author contributions

SZ: Writing – review & editing, Writing – original draft, Software, Formal analysis, Data curation, Conceptualization. XC: Writing – original draft, Methodology. QD: Writing – review & editing, Resources, Project administration, Data curation. HG: Writing – original draft, Resources. LS: Writing – original draft, Software. QZ: Writing – original draft, Supervision. YG: Writing – original draft, Validation.

## References

[B1] Alan K. K. Claus B. David D. B. Aimée T. C. Markus R. (2008). Consequences of more extreme precipitation regimes for terrestrial ecosystems. BioScience 58, 811–821. doi: 10.1641/B580908

[B2] Ansley R. J. Boutton T. W. Jacoby P. W. (2014). Root biomass and distribution patterns in a semi-arid mesquite savanna, responses to long-term rainfall manipulation. Rangeland Ecol. Manage. 67, 206–218. doi: 10.2111/REM-D-13-00119.1

[B3] Ashraf M. Harris P. J. C. (2004). Potential biochemical indicators of salinity tolerance in plants. J. Plant Physiol. 166 (1), 3–16. doi: 10.1016/j.plantsci.2003.10.024

[B4] Bai Y. Wu J. Xing Q. Pan Q. Huang J. Yang D.. (2008). Primary production and rain use efficiency across a precipitation gradient on the Mongolia plateau. Ecology 89, 2140–2153. doi: 10.1890/07-0992.1 18724724

[B5] Blumstein M. Hopkins R. (2021). Adaptive variation and plasticity in non-structural carbohydrate storage in a temperate tree species. Plant Cell Environ. Plant 44, 2494–2505. doi: 10.1111/pce.13959 33244757

[B6] Dai M. Xiao Y. Wang T. Xu J. Wang Y. (2022). Influence of N:P ratio of water on ecological stoichiometry of vallisneria natans and hydrilla verticillata. Water 14, 1263. doi: 10.3390/w14081263

[B7] Dietze M. C. Sala A. Carbone M. S. Czimczik C. I. Mantooth J. A. Richardson A. D.. (2014). Nonstructural carbon in woody plants. Annu. Rev. Plant Biol. 65, 667–687. doi: 10.1146/annurev-arplant-050213-040054 24274032

[B8] Diffenbaugh N. S. Giorgi F. (2012). Climate change hotspots in the CMIP5 global climate model ensemble. Climatic Change 114, 813–822. doi: 10.1007/s10584-012-0570-x 24014154 PMC3765072

[B9] Dong L. J. Sang W. G. (2012). Effects of simulated warming and precipitation change on seedling emergence and growth of Quercus mongolica in Dongling Mountain, Beijing, China. Chin. J. Plant Ecol. 36 (8), 819–830. doi: 10.3724/SP.J.1258.2012.00819

[B10] Dong Y. H. Liu B. B. Zhang X. Liu X. N. Ai X. Z. Li Q. M. (2015). Responses of non-structural carbohydrate metabolism of cucumber seedlings to drought stress and doubled CO_2_ concentration. Ying yong sheng tai xue bao = J. Appl. Ecol. 26, 53–60. doi: 10.13287/j.1001-9332.20141021.007 25985653

[B11] Du Y. Han Y. Wang C. K. (2014). The influence of drought on non-structural carbohydrates in the needles and twigs of Larix gmelinii. Acta Ecologica Sin. 34, 6090–6100. doi: 10.5846/stxb201401260198

[B12] Duan Y. Y. Wang Z. L. Wang S. J. (2019). Analysis and risk study of water storage model of Kunming reservoir based on rainfall. Yunnan Geographic Environ. Res. 31, 13–19. doi: 1001-7852(2019)02-0013-07

[B13] Elser J. J. Fagan W. F. Kerkhoff A. J. Swenson N. G. Enquist B. J. (2010). Biological stoichiometry of plant production: metabolism, scaling and ecological response to global change. New Phytol. 186, 593–608. doi: 10.1111/j.1469-8137.2010.03214.x 20298486

[B14] Elser J. J. Sterner R. W. Gorokhova E. Fagan W. F. Markow T. A. Cotner J. B.. (2000). Biological stoichiometry from genes to ecosystems. Ecol. Lett. 3, 540–550. doi: 10.1111/j.1461-0248.2000.00185.x

[B15] Falster D. S. Warton D. I. Wright I. J. (2006). User’s guide to SMATR: Standardised Major Axis Tests & Routines Version 2.0. Available online at: http://www.bio.mq.edu.au/ecology/SMATR/SMATR_users_guide.pdf.

[B16] Fay P. A. Carlisle J. D. Knapp A. K. Blair J. M. Collins S. L. (2000). Altering rainfall timing and quantity in a mesic grassland ecosystem: Design and performance of rainfall manipulation shelters. Ecosystems 3, 308–319. doi: 10.1007/s100210000028

[B17] Fay P. A. Carlisle J. D. Knapp A. K. Blair J. M. Collins S. L. (2003). Productivity responses to altered rainfall patterns in a C4-dominated grassland. Oecologia 137, 245–251. doi: 10.1007/s00442-003-1331-3 12845518

[B18] Gao J. Li X. B. Li X. W. (2015). Drought resistance of eight major tree species in semi-dry thermal stone desertification management in southeast Yunnan. J. Southwest Forestry Univ. 35, 1–10. doi: 10.11929/j.issn.2095-1914.2015.02.001

[B19] Gao Y. H. Jiang C. Y. Hu Y. G. Wang X. F. (2021). Effects of different fertilizer treatments on nutrient uptake and partitioning in organic astragalus. Chin. J. Ecol. Agric. (in English) 29, 453–464. doi: 10.13930/j.cnki.cjea.200631

[B20] Gao Y. Zhu B. Yu G. Chen W. He N. Wang T.. (2014). Coupled effects of biogeochemical and hydrological processes on C, N, and P export during extreme rainfall events in a purple soil watershed in southwestern China. J. hydrology 511, 692–702. doi: 10.1016/j.jhydrol.2014.02.005

[B21] Gheyret G. Zhang H. T. Guo Y. Liu T. Y. Bai Y. H. Li S.. (2021). Radial growth response of trees to seasonal soil humidity in a subtropical forest. Basic Appl. Ecol. 55, 74–86. doi: 10.1016/j.baae.2021.02.015

[B22] Gibson S. I. (2005). Control of plant development and gene expression by sugar signaling. Curr. Opin. Plant Biol. 8, 93–102. doi: 10.1016/j.pbi.2004.11.003 15653406

[B23] Goldstein L. J. Suding K. N. (2014). Intra-annual rainfall regime shifts competitive interactions between coastal sage scrub and invasive grasses. Ecology 95, 425–435. doi: 10.1890/12-0651.1 24669735

[B24] Goodsman D. W. Lieffers V. J. Landhäusser S. M. Erbilgin N. (2010). Fertilization of lodgepole pine trees increased diameter growth but reduced root carbohydrate concentrations. For. Ecol. Manage. 260, 1914–1920. doi: 10.1016/j.foreco.2010.08.041

[B25] Han W. X. Fang J. Y. Reich P. B. Ian Woodward F. Wang Z. H. (2011). Biogeography and variability of eleven mineral elements in plant leaves across gradients of climate, soil and plant functional type in China. Ecol. Lett. 14, 788–796. doi: 10.1111/j.1461-0248.2011.01641.x 21692962

[B26] Harper C. W. Blair J. M. Fay P. A. Knapp A. K. Carlisle J. D. (2005). Increased rainfall variability and reduced rainfall amount decreases soil CO_2_ flux in a grassland ecosystem. Global Change Biol. 11, 322–334. doi: 10.1111/j.1365-2486.2005.00899.x

[B27] Hartmann H. Trumbore S. (2016). Understanding the roles of nonstructural carbohydrates in forest trees-from what we can measure to what we want to know. New Phytol. 211, 386–403. doi: 10.1111/nph.13955 27061438

[B28] He Q. Silliman B. R. Liu Z. Cui B. (2017). Natural enemies govern ecosystem resilience in the face of extreme droughts. Ecol. Lett. 20, 194–201. doi: 10.1111/ele.12721 28058801

[B29] He Z. Xu Y. P. Zhang X. M. (2012). Effect of different concentrations of gibberellin on the germination of Fraxinus malacophylla seeds. Shandong Forestry Sci. Technol. 42, 20–23. doi: 1002-2724(2012)02-0020-04

[B30] Heisler-white J. L. Blair J. M. Kelly E. F. Harmoney K. Knapp A. K. (2009). Contingent productivity responses to more extreme rainfall regimes across a grassland biome. Global Change Biol. 15, 2894–2904. doi: 10.1111/j.1365-2486.2009.01961.x

[B31] Hertel D. Strecker T. Müller-Haubold H. Leuschner C. (2013). doi: 10.1111/1365-2745.12124

[B32] Hoegh-Guldberg O. Jacob D. Taylor M. Guillén Bolaños T. Bindi M. Brown S.. (2019). The human imperative of stabilizing global climate change at 1.5°C. Sci. (New York N.Y.) 365. doi: 10.1126/science.aaw6974 31604209

[B33] Huang C. L. Chen Q. (2014). Afforestation technology of Fraxinus malacophylla in semi-arid subtropical rocky desertification area. For. Sci. Technol. 39(12), 20–22. doi: 10.13456/j.cnki.1ykt.2014.12.007

[B34] Irannejad H. Shahbazian N. (2004). Filed crops tolerance to stress (Iran: University of Tehran Press).

[B35] Jentsch A. Kreyling J. Beierkuhnlein C. (2007). A new generation of climate-change experiments: events, not trends. Front. Ecol. Environ. 5, 365–374. doi: 10.1890/1540-9295

[B36] Jia W. Zhang J. H. (2008). Stomatal movements and long-distance signaling in plants. Plant Signaling Behav. 3, 772–777. doi: 10.4161/psb.3.10.6294 PMC2634372 19513229

[B37] Kardol P. Campany C. E. Souza L. Norby R. J. Weltzin J. F. Classen A. T. (2010). Climate change effects on plant biomass alter dominance patterns and community evenness in an experimental old-field ecosystem. Global Change Biol. 16, 2676–2687. doi: 10.1111/j.1365-2486.2010.02162.x

[B38] Kimball S. Angert A. L. Huxman T. E. Venable D. L. (2010). Contemporary climate change in the Sonoran Desert favors cold-adapted species. Global Change Biol. 16, 1555–1565. doi: 10.1111/j.1365-2486.2009.02106.x

[B39] Klausmeier C. A. Litchman E. Daufresne T. Levin S. A. (2004). Optimal nitrogen-to-phosphorus stoichiometry of phytoplankton. Nature 429, 171–174. doi: 10.1038/nature02454 15141209

[B40] Knapp A. K. Fay P. A. Blair J. M. Collins S. L. Smith M. D. Carlisle J. D.. (2002). Rainfall variability, carbon cycling, and plant species diversity in a mesic grassland. Science 298, 2202–2205. doi: 10.1126/science.1076347 12481139

[B41] Knapp A. K. Smith M. D. (2001). Variation among biomes in temporal dynamics of aboveground primary production. Science 291, 481–484. doi: 10.1126/science.291.5503.481 11161201

[B42] Kopittke P. M. Dalal R. C. Finn D. Menzies N. W. (2016). Global changes in soil stocks of carbon, nitrogen, phosphorus, and sulphur as influenced by long-term agricultural production. Global Change Biol. 23, 2509–2519. doi: 10.1111/gcb.13513 27670741

[B43] Körner C. (2003). Carbon limitation in trees. J. Ecol. 91, 4–17. doi: 10.1046/j.1365-2745.2003.00742.x

[B44] Latt C. R. Nair P. K. R. Kang B. T. (2001). Reserve carbohydrate levels in the boles and structural roots of five multipurpose tree species in a seasonally dry tropical climate. For. Ecol. Manage. 146, 145–158. doi: 10.1016/s0378-1127(00)00456-4

[B45] Li B. F. (2016). Spatial-temporal distribution characteristics of annual precipitation in Kunming (China: Kunming Branch of Yunnan Provincial Bureau of Hydrology and Water Resources), 123–133.

[B46] Li Y. C. Chen S. L. Yue Y. D. Liu Y. F. Guo Z. W. Yang Q. P. (2017). Effect of continuous flooding stress on nutrient element distribution patterns in Phyllostachys rivalis modules. Acta Ecologica Sin. 37, 3482–3493. doi: 10.5846/stxb201602150282

[B47] Li N. Sun T. Mao Z. J. (2014). Effects of long-term high-temperature stress on the biomass and non-structure carbohydrates of Pinus sylves-tris var. mongolica seedlings. Bull. Botanical Res. 34, 212–218. doi: 10.7525/j.issn.1673-5102.2014.02.012

[B48] Li X. W. (2005). Domestication and application of wild species of Fraxinus malacophylla in stone desertification management. Yunnan Province, Southwest Forestry College, 01–31.

[B49] Liu L. Ge J. L. Shu H. W. Zhao C. M. Xu W. T. Shen G. Z.. (2019). C, N and P stoichiometric ratios in mixed evergreen and deciduous broadleaved forests in Shennongjia, China. Chin. J. Plant Ecol. 43, 482–489. doi: 10.17521/cjpe.2019.0064

[B50] Liu M. H. Qiu N. X. Chen Y. L. Huang J. L. (2020). Study on change characteristics of non effective precipitation days in southwest China from 1960 to 2016. Pearl river 41, 21–29. doi: 10.3969/j.issn.1001-9235.2020.04.004

[B51] Loewe A. Einig W. Shi L. Dizengremel P. Hampp R. (2000). Mycorrhiza formation and elevated CO2 both increase the capacity for sucrose synthesis in source leaves of spruce and aspen. New Phytol. 145, 565–574. doi: 10.1046/j.1469-8137.2000.00598.x 33862899

[B52] Loik M. E. Breshears D. D. Lauenroth W. K. Belnap J. (2004). A multi-scale perspective of water pulses in dryland ecosystems:climatology and ecohydrology of the western USA. Oecologia 141 (2), 269–281. doi: 10.1007/s00442-004-1570-y 15138879

[B53] Luo Y. Peng Q. Li K. Gong Y. Liu Y. Han W. (2021). Patterns of nitrogen and phosphorus stoichiometry among leaf, stem and root of desert plants and responses to climate and soil factors in Xinjiang, China. Catena 199, 105100. doi: 10.1016/j.catena.2020.105100

[B54] Lüthi D. Floch M. Bereiter B. Blunier T. Barnola J. M. Siegenthaler U.. (2008). High-resolution carbon dioxide concentration record 650,000–800,000 years before present. Nature 453, 379–382. doi: 10.1038/nature06949 18480821

[B55] Ma R. An S. Huang Y. (2017). C, N and P stoichiometry characteristics of different-aged Robinia pseudoacacia plantations on the Loess Plateau, China. Chin. J. Appl. Ecol. 28, 2787–2793. doi: 10.13287/j.1001-9332.201709.020

[B56] Mao R. Chen H. M. Zhang X. H. Shi F. X. Song C. C. (2016). Effects of P addition on plant C: N: P stoichiometry in an N-limited temperate wetland of Northeast China. Sci. Total Environ. 559, 1–6. doi: 10.1016/j.scitotenv.2016.03.158 27045368

[B57] Martin A. R. Doraisami M. Thomas S. C. (2018). Global patterns in wood carbon concentration across the world’s trees and forests. Nat. Geosci. 11, 915–920. doi: 10.1038/s41561-018-0246-x

[B58] Masson-delmotte V. Zhai P. PiraniI A. (2021). Climate change 2021: The physical science basis: summary for policymakers: working group I contribution to the sixth assessment report of the intergovernmental panel on climate change (Geneva, Switzerland: IPCC). doi: 10.3410/f.740620545.793587812

[B59] McDowell N. G. (2011). Mechanisms linking drought, hydraulics, carbon metabolism, and vegetation mortality. Plant Physiol. 155, 1051–1059. doi: 10.1104/pp.110.170704 21239620 PMC3046567

[B60] Myers J. A. Kitajima K. (2007). Carbohydrate storage enhances seedling shade and stress tolerance in a neotropical forest. J. Ecol. 95, 383–395. doi: 10.1111/j.1365-2745.2006.01207.x

[B61] Peters R. L. Steppe K. Cuny H. E. De J. W. Pauw D. Frank D.. (2020). Turgor – a limiting factor for radial growth in mature conifers along an elevational gradient. New Phytol. 229, 213–229. doi: 10.1111/nph.16872 32790914

[B62] Poorter L. Kitajima K. (2007). Carbohydrate storage and light requirements of tropical moist and dry forest tree species. Ecology 88, 1000–1011. doi: 10.1890/06-0984 17536715

[B63] Post A. K. Knapp A. K. (2020). The importance of extreme rainfall events and their timing in a semi-arid grassland. J. Ecol. 108, 2431–2443. doi: 10.1111/1365-2745.13478

[B64] Prommer J. Walker T. W. Wanek W. Braun J. Zezula D. Hu Y.. (2019). Increased microbial growth, biomass, and turnover drive soil organic carbon accumulation at higher plant diversity. Global Change Biol. 26, 669–681. doi: 10.1111/gcb.14777 PMC7027739 31344298

[B65] Qu F. Yu J. Du S. Li Y. Lv X. Ning K.. (2014). Influences of anthropogenic cultivation on C, N and P stoichiometry of reed-dominated coastal wetlands in the Yellow River Delta. Geoderma 235, 227–232. doi: 10.1016/j.geoderma.2014.07.009

[B66] Rastetter E. B. Kwiatkowski B. L. Kicklighter D. W. Barker P. A. Genet H. Nippert J. B.. (2022). N and P constrain C in ecosystems under climate change: Role of nutrient redistribution, accumulation, and stoichiometry. Ecol. Appl. 32, e2684. doi: 10.1002/eap.2684 35633204 PMC10078338

[B67] Raven J. A. (2015). Interactions between nitrogen and phosphorus metabolism. Annu. Plant Rev. Volume 48: Phosphorus Metab. Plants 48, 187–214. doi: 10.1002/9781118958841.ch7

[B68] Rosas T. Galiano L. Ogaya R. Peñuelas J. Martínez-Vilalta J. (2013). Dynamics of non-structural carbohydrates in three Mediterranean woody species following long-term experimental drought. Front. Plant Sci. 4. doi: 10.3389/fpls.2013.00400 PMC3795346 24130568

[B69] Rouphael Y. Cardarelli M. Schwarz D. Franken P. Colla G. (2012). Effects of drought on nutrient uptake and assimilation in vegetable crops. Plant responses to drought stress: morphological to Mol. features, 171–195. doi: 10.1007/978-3-642-32653-0_7

[B70] Sardans J. Rivas-Ubach A. Penuelas J. (2012). The C: N: P stoichiometry of organisms and ecosystems in a changing world: A review and perspectives. Perspect. Plant Ecology Evol. Systematics 14, 33–47. doi: 10.1016/j.ppees.2011.08.002

[B71] Shi L. J. Li Q. K. Fu X. L. Kou L. Dai X. Q. Wang H. M. (2021). Foliar, root and rhizospheric soil C: N: P stoichiometries of overstory and understory species in subtropical plantations. Catena 198, 105020. doi: 10.1016/j.catena.2020.105020

[B72] Sierra C. A. Malghani S. Loescher H. W. (2017). Interactions among temperature, moisture, and oxygen concentrations in controlling decomposition rates in a boreal forest soil. Biogeosciences 14, 703–710. doi: 10.5194/bg-14-703-2017

[B73] Smith M. D. (2011a). An ecological perspective on extreme climatic events: a synthetic definition and framework to guide future research. J. Ecol. 99, 656–663. doi: 10.1111/j.1365-2745.2011.01798.x

[B74] Smith M. D. (2011b). The ecological role of climate extremes: current understanding and future prospects. J. Ecol. 99, 651–655. doi: 10.1111/j.1365-2745.2011.01833.x

[B75] Sponseller R. A. (2007). Precipitation pulses and soil CO_2_ flux in a Sonoran Desert ecosystem. Global Change Biol. 13, 426–436. doi: 10.1111/j.1365-2486.2006.01307.x

[B76] Sun J. Q. Xiong W. B. Li Y. Q. Cai T. R. Yu H. (2021). Stoichiometric characteristics of carbon, nitrogen and phosphorus in fine root of plants with differentlife forms in China and theirinfluencing factors. Prot. For. Sci. Technol. 4, 28–32. doi: 10.13601/j.issn.1005-5215.2021.04.010

[B77] Swemmer A. M. Knapp A. K. Snyman H. A. (2007). Intra-seasonal precipitation patterns and above-ground productivity in three perennial grasslands. J. Ecol. 95, 780–788. doi: 10.1111/j.1365-2745.2007.01237.x

[B78] Tan M. X. Gan D. H. Wei L. X. Pan Y. M. Tang S. Q. Wang H. S. (2011). Isolation and characterization of pigment from Cinnamomum burmannii’peel. Food Res. Int. 44, 2289–2294. doi: 10.1016/j.foodres.2010.05.022

[B79] Tan X. Q. Guo L. J. Zheng W. Tan C. H. (2013). Study on chemical components of Fraxinus malacophylla (I). China Pharm. 24, 4081–4083. doi: 10.6039/j.issn.1001-0408.2013.43.17

[B80] Tang Z. Xu W. Zhou G. Bai Y. Li J. Tang X.. (2018). Patterns of plant carbon, nitrogen, and phosphorus concentration in relation to productivity in China’s terrestrial ecosystems. Proc. Natl. Acad. Sci. U. S. A. 115, 4033–4038. doi: 10.1073/pnas.1700295114 29666316 PMC5910803

[B81] Thakur M. P. Milcu A. Manning P. Niklaus P. A. Roscher C. Power S.. (2015). Plant diversity drives soil microbial biomass carbon in grasslands irrespective of global environmental change factors. Global Change Biol. 21, 4076–4085. doi: 10.1111/gcb.13011 26118993

[B82] Thalmann M. Santelia D. (2017). Starch as a determinant of plant fitness under abiotic stress. New phytologist,214 3), 943–951. doi: 10.1111/nph.14491 28277621

[B83] Vile D. Pervent M. Belluau M. Vasseur F. Bresson J. Muller B.. (2012). Arabidopsis growth under prolonged high temperature and water deficit: independent or interactive effects? Plant Cell Environ. 35, 702–718. doi: 10.1111/j.1365-3040.2011.02445.x 21988660

[B84] Walck J. L. Hidayati S. N. Dixon K. W. Thompson K. E. N. Poschlod P. (2011). Climate change and plant regeneration from seed. Global Change Biol. 17, 2145–2161. doi: 10.1111/j.1365-2486.2010.02368.x

[B85] Wang G. L. Fahey T. J. Xue S. Liu F. (2013). Root morphology and architecture respond to N addition in Pinus tabuliformis, West China. Oecologia 171, 583–590. doi: 10.1007/s00442-012-2441-6 22948279

[B86] Wang K. Lei H. Xia Y. Yu G. Q. (2017). Responses of non-structural carbohydrates of poplar seedlings to increased precipitation and nitrogen addition. Ying Yong Sheng tai xue bao=The J. Appl. Ecol. 28, 399–407. doi: 10.13287/j.1001-9332.201702.012 29749146

[B87] Wang X. Lou W. T. Yu Q. (2014). Effects of nutrient addition on nitrogen, phosphorus and non-structural carbohydrates concentrations in leaves of dominant plant species in a semiarid steppe. Chin. J. Ecol. 33, 1795–1802. doi: 10.13292/j.1000-4890.20140422.028

[B88] Wang K. Pang Y. Y. Zhang R. S. Shen C. Song L. N. (2021). Allocation of non-structural carbohydrates of different aged Pinus sylvestris var. mongolica sandland. Chin. J. Ecol. 40, 1264–1274. doi: 10.13292/j.1000-4890.202105.022

[B89] Wang X. Zhou X. Sun Z. (2005). Research advances in the relationship between alpine timberline and climate change. Chin. J. Ecol. 24, 301–305. doi: 10.13292/j.1000-4890.2005.0257

[B90] Warton D. I. Weber N. C. (2002). Common slope tests for bivariate errors-in-variables models. Biometrical Journal: J. Math. Methods Biosci. 44, 161–174. doi: 10.1002/1521-4036(200203)44:2<161::AID-BIMJ161>3.0.CO;2-N

[B91] Weiss J. L. Gutzler D. S. Coonrod J. E. A. Dahm C. N. (2004). Longterm vegetation monitoring with NDVI in a diverse semi-arid setting, central New Mexico, USA. J. Arid Environments 58, 249–272. doi: 10.1016/j.jaridenv.2003.07.001

[B92] Westoby M. Falster D. S. Moles A. T. Vesk P. A. Wright I. J. (2002). Plant ecological strategies: some leading dimensions of variation between species. Annu. Rev. Ecol. systematics 33, 125–159. doi: 10.1146/annurev.ecolsys.33.010802.150452

[B93] Witek-Krowiak A. Szafran R. G. Modelski S. (2011). Biosorption of heavy metals from aqueous solutions onto peanut shell as a low-cost biosorbent. Desalination 265, 126–134. doi: 10.1016/j.desal.2010.07.042

[B94] Worm B. Lotze H. K. (2021). Marine biodiversity and climate change. In Climate Change pp, 445–464). doi: 10.1016/b978-0-12-821575-3.00021-9

[B95] Wu Y. L. Zhao B. Li Q. Kong F. L. Du L. J. Fang Z.. (2019). Non-structural carbohydrates in maize with different nitrogen tolerance are affected by nitrogen addition. PloS One 14, e0225753. doi: 10.1371/journal.pone.0225753 31805168 PMC6894874

[B96] Wurzburger N. Wright S. J. (2015). Fine-root responses to fertilization reveal multiple nutrient limitation in a lowland tropical forest. Ecology 96, 2137–2146. doi: 10.1890/14-1362.1 26405739

[B97] Xia Z. Y. He Z. Xu Y. P. (2016). Effects of fertilization with nitrogen, phosphorus and potassium ratios and hormone treatments on the growth of Fraxinus malacophylla. Forestry Survey Plann. 41 (3), 82–86. doi: 10.3969/j.issn.1671-3168.2016.03.019

[B98] Xie T. Shan L. Zhang W. (2022). N addition alters growth, non-structural carbohydrates, and C: N: P stoichiometry of Reaumuria soongorica seedlings in Northwest China. Sci. Rep. 12, 15390. doi: 10.1038/s41598-022-19280-8 36100614 PMC9470663

[B99] Yang X. Cui D. Zhao Y. Yan J. J. Zhang S. S. Liu Y. Y. (2021). Stoichiometric characteristics of Sophora alopecuroides roots in different habitats of Yili River valley and their relationship with soil physicochemical factors. Chin. J. Ecol. 40, 1305–1312. doi: 10.13292/j.1000-4890.202105.034

[B100] Yang Y. Liu B. R. An S. S. (2018). Ecological stoichiometry in leaves, roots, litters and soil among different plant communities in a desertified region of Northern China. Catena 166, 328–338. doi: 10.1016/j.catena.2018.04.018

[B101] Yang B. Peng C. H. Zhang X. Liu W. G. Duan M. (2019). Effects of drought stress on leaf nitrogen, photosynthetic rate and non-structural carbohydrates of Robinia pseudoacacia L. seedlings. Chin. J. Appl. Environ. Biol. 25, 1261–1269. doi: 10.19675/j.cnki.1006-687x.2019.03011

[B102] Ye Y. Liang X. Chen Y. Li L. Ji Y. Zhu C. (2014). Carbon, nitrogen and phosphorus accumulation and partitioning, and C: N: P stoichiometry in late-season rice under different water and nitrogen managements. PloS One 9, e101776. doi: 10.1371/journal.pone.0101776 24992006 PMC4081737

[B103] Yin J. J. Guo D. L. He S. Y. Zhang L. (2009). Non-structural carbohydrate, N, and P allocation patterns of two temperate tree species in a semi-arid region of Inner Mongolia. Acta Scientiarum Naturalium Universitatis Pekinensis 45, 519–527. doi: 10.13209/j.0479-8023.2009.077

[B104] Yu L. Wang C. K. Wang X. C. (2011). Allocation of nonstructural carbohydrates for three temperate tree species in Northeast China. Chin. J. Plant Ecol. 35, 1245–1255. doi: 10.3724/sp.j.1258.2011.01245

[B105] Yuan Z. Y. Chen H. Y. H. Reich P. B. (2011). Global-scale latitudinal patterns of plant fine-root nitrogen and phosphorus. Nat. Commun. 2, 344. doi: 10.1038/ncomms1346 21673665

[B106] Yue X. Zhang T. Zhao X. Liu X. Ma Y. (2016). Effects of rainfall patterns on annual plants in Horqin Sandy Land, Inner Mongolia of China. J. Arid Land 8, 389–398. doi: 10.1007/s40333-016-0044-5

[B107] Zechmeister B. S. Keiblinger K. M. Mooshammer M. Peuelas J. Richter A. Sardans J. (2015). The application of ecological stoichiometry to plant-microbial-soil organic matter transformations. Ecol. Monogr. 85, 133–155. doi: 10.1890/14-0777.1

[B108] Zhang J. H. He N. P. Liu C. C. Xu L. Yu Q. Yu G. R. (2018). Allocation strategies for nitrogen and phosphorus in forest plants. Oikos 127, 1506–1514. doi: 10.1111/oik.05517

[B109] Zhang W. Liu W. C. Xu M. P. Deng J. Han X. H. Yang G. H. (2019). Response of forest growth to C: N: P stoichiometry in plants and soils during Robinia pseudoacacia afforestation on the loess plateau, China. Geoderma 337, 280–289. doi: 10.1016/j.geoderma.2018.09.042

[B110] Zhang H. Wang C. Wang X. (2015). Within-crown variation in concentrations of non-structural carbohydrates of five temperate tree species. Acta Ecologica Sin. 35, 6496–6506. doi: 10.5846/stxb201402080223

[B111] Zhang W. P. Zhao L. Larjavaara M. Morris E. C. Sterck F. J. Wang G. X. (2020). Height-diameter allometric relationships for seedlings and trees across China. Acta Oecologica 108, 103621. doi: 10.1016/j.actao.2020.103621

[B112] Zhang J. Zuo X. Zhao X. Ma J. Medina-Roldán E. (2020). Effects of rainfall manipulation and nitrogen addition on plant biomass allocation in a semiarid sandy grassland. Sci. Rep. 10, 9026. doi: 10.1038/s41598-020-65922-0 32493956 PMC7270118

[B113] Zhao B. Jia X. Yu N. Murray J. D. Yi K. Wang E. (2023). Microbe-dependent and independent nitrogen and phosphate acquisition and regulation in plants. New Phytol. doi: 10.1111/nph.19263 37715479

[B114] Zheng Y. P. Wang H. X. Lou X. (2014). Changes of non-structural carbohydrates and its impact factors in trees: A review. Yingyong Shengtai Xuebao 25, 1188–1196. 25011317

[B115] Zhu H. Zhao J. Gong L. (2021). The morphological and chemical properties of fine roots respond to nitrogen addition in a temperate schrenk’s spruce (Picea schrenkiana) forest. Sci. Rep. 11, 3839. doi: 10.1038/s41598-021-83151-x 33589690 PMC7884734

